# *ADAM17* variant causes hair loss via ubiquitin ligase TRIM47–mediated degradation

**DOI:** 10.1172/jci.insight.177588

**Published:** 2024-05-21

**Authors:** Xiaoxiao Wang, Chaolan Pan, Luyao Zheng, Jianbo Wang, Quan Zou, Peiyi Sun, Kaili Zhou, Anqi Zhao, Qiaoyu Cao, Wei He, Yumeng Wang, Ruhong Cheng, Zhirong Yao, Si Zhang, Hui Zhang, Ming Li

**Affiliations:** 1 Department of Dermatology, Xinhua Hospital, and; 2 Institute of Dermatology, Shanghai Jiaotong University School of Medicine, Shanghai, China.; 3 Department of Dermatology, Anhui Provincial Children’s Hospital, Hefei, China.; 4 Department of Dermatology, Henan Provincial People’s Hospital, Zhengzhou University People’s Hospital, and Henan University People’s Hospital, Zhengzhou, China.; 5 Department of Dermatology, The Children’s Hospital of Fudan University, Shanghai, China.; 6 NHC Key Laboratory of Glycoconjugate Research, Department of Biochemistry and Molecular Biology, School of Basic Medical Sciences, Fudan University, Shanghai, China.

**Keywords:** Dermatology, Genetics, Genetic diseases, Genetic variation, Ubiquitin-proteosome system

## Abstract

Hypotrichosis is a genetic disorder characterized by a diffuse and progressive loss of scalp and/or body hair. Nonetheless, the causative genes for several affected individuals remain elusive, and the underlying mechanisms have yet to be fully elucidated. Here, we discovered a dominant variant in a disintegrin and a metalloproteinase domain 17 (*ADAM17*) gene caused hypotrichosis with woolly hair. *Adam17* (p.D647N) knockin mice mimicked the hair abnormality in patients. *ADAM17* (p.D647N) mutation led to hair follicle stem cell (HFSC) exhaustion and caused abnormal hair follicles, ultimately resulting in alopecia. Mechanistic studies revealed that ADAM17 binds directly to E3 ubiquitin ligase tripartite motif-containing protein 47 (TRIM47). *ADAM17* variant enhanced the association between ADAM17 and TRIM47, leading to an increase in ubiquitination and subsequent degradation of ADAM17 protein. Furthermore, reduced ADAM17 protein expression affected the Notch signaling pathway, impairing the activation, proliferation, and differentiation of HFSCs during hair follicle regeneration. Overexpression of Notch intracellular domain rescued the reduced proliferation ability caused by *Adam17* variant in primary fibroblast cells.

## Abstract 2

An autosomal dominant hypotrichosis-associated ADAM17 variant increases the interaction between ADAM17 and TRIM47, leading to heightened autoubiquitination and subsequent ADAM17 degradation.

## Graphical abstract

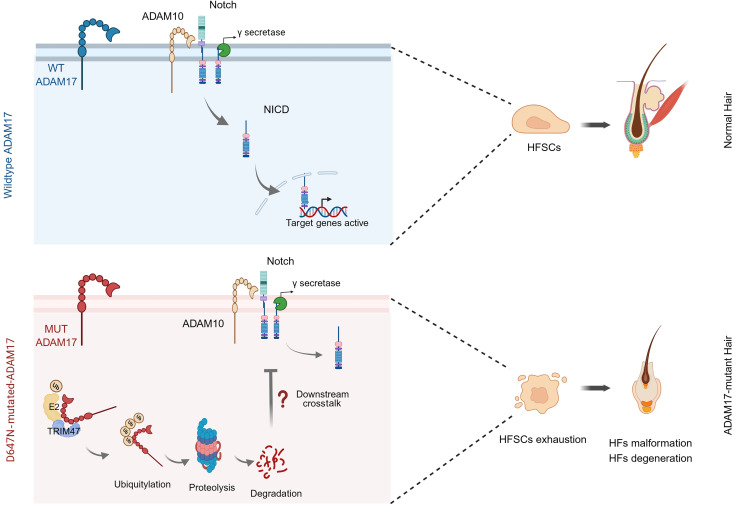

## Introduction

Human hair, especially human scalp hair, has important ornamental functions that are essential for social communication and sense of well-being. Unwanted hair loss poses psychosocial distress to affected individuals and affects their quality of life (1). A comprehensive survey conducted on individuals with hair loss revealed that they are more prone to experiencing anxiety, depression, and sleep disorders (2). Additionally, a concerning increase in suicide rates has been found among individuals suffering from hair loss (3, 4). Congenital hypotrichosis (HYPT) is a hair disorder marked by sparse or complete absence of hair on the scalp and/or other body parts. Occasionally, some cases accompanied by tightly coiled, curled, or woolly hair are called autosomal recessive woolly hair (ARWH) or autosomal dominant woolly hair (ADWH) depending on their congenital pattern (5, 6). *Keratin* (KRT) *74* and *KRT71* mutations are known to cause nonsyndromic ADWH1 (OMIM#613981) and ADWH2 (OMIM#615896), respectively (7, 8). Nevertheless, the causative genes for several affected individuals are still unidentified, and the underlying mechanisms have not been fully comprehended.

Adisintegrin and ametalloproteinase domain 17 (ADAM17), a type I transmembrane metallopeptidase, is responsible for shedding the ectodomains of over 90 substrates, including cytokines (such as TNF-α), cytokine receptors (such as IL-6R and TNFR), and adhesion proteins (9). It functions as a molecular switch to regulate both inflammation and tissue regeneration and is linked to various inflammatory diseases, such as Alzheimer’s disease, inflammation-related atherosclerosis, and rheumatoid arthritis (10–12). In addition, there is compelling evidence supporting the critical involvement of ADAM17 in many cancers and as a potential therapeutic target (13–15). Even though ADAM17 can cleave multiple substrates in vitro or in cell-free assays, only some of them have a substantial impact on development and disease in vivo. For instance, in skin, deletion of ADAM17 in keratinocytes stimulates atopic dermatitis, driven by Th2 and/or Th17 response(s), due to a selective hindrance in the recruitment of the transcription factor c-Fos to the Th2-polarizing cytokine TSLP (16). ADAM17 regulates epidermal growth factor receptor (EGFR) ligand-dependent terminal keratinocyte differentiation, thus preserving the skin barrier (17). Additionally, ADAM17 is also involved in the development of the hair follicle inner root sheath (IRS) and the establishment of hair follicle stem cell (HFSC) niche (18, 19). Further exploration is necessary to fully understand the impact of ADAM17 on hair follicle development.

Tripartite motif-containing protein 47 (TRIM47) functions as an E3 ubiquitin ligase and is involved in numerous biological processes, including tumorigenesis, cerebral ischemia/reperfusion injury, and endothelial inflammation (20–24). The interaction between E3 ubiquitin ligase and the target protein is a core step in ubiquitin–proteasome system–mediated protein degradation (25). TRIM47 aggravates lipopolysaccharide-induced acute lung injury through the K63-linked ubiquitination of TRAF2 (26). TRIM47 promotes tumorigenesis by facilitating the ubiquitination and subsequent degradation of SMAD4, FBP1, and PKC-ε/PKD3 (21, 23, 27). Nevertheless, the pathologic and clinical roles of TRIM47 in hair follicles remain unclear.

In this study, we identified that a variant in the conserved ADAM17 dynamic interaction sequence (CANDIS) domain of *ADAM17* caused autosomal dominant hypotrichosis with woolly hair. An *Adam17*-knockin mouse model mimicked the hair abnormality in patients. We discovered that TRIM47, an E3 ubiquitin ligase, interacted with the mutant ADAM17, promoting its ubiquitination and ultimate protein degradation. Eventually, reduced ADAM17 expression led to dysfunction in the Notch signaling pathway, which in turn contributed to hair follicle malformation.

## Results

### ADAM17 variant leads to autosomal dominant hypotrichosis with woolly hair.

**Figure 1:**
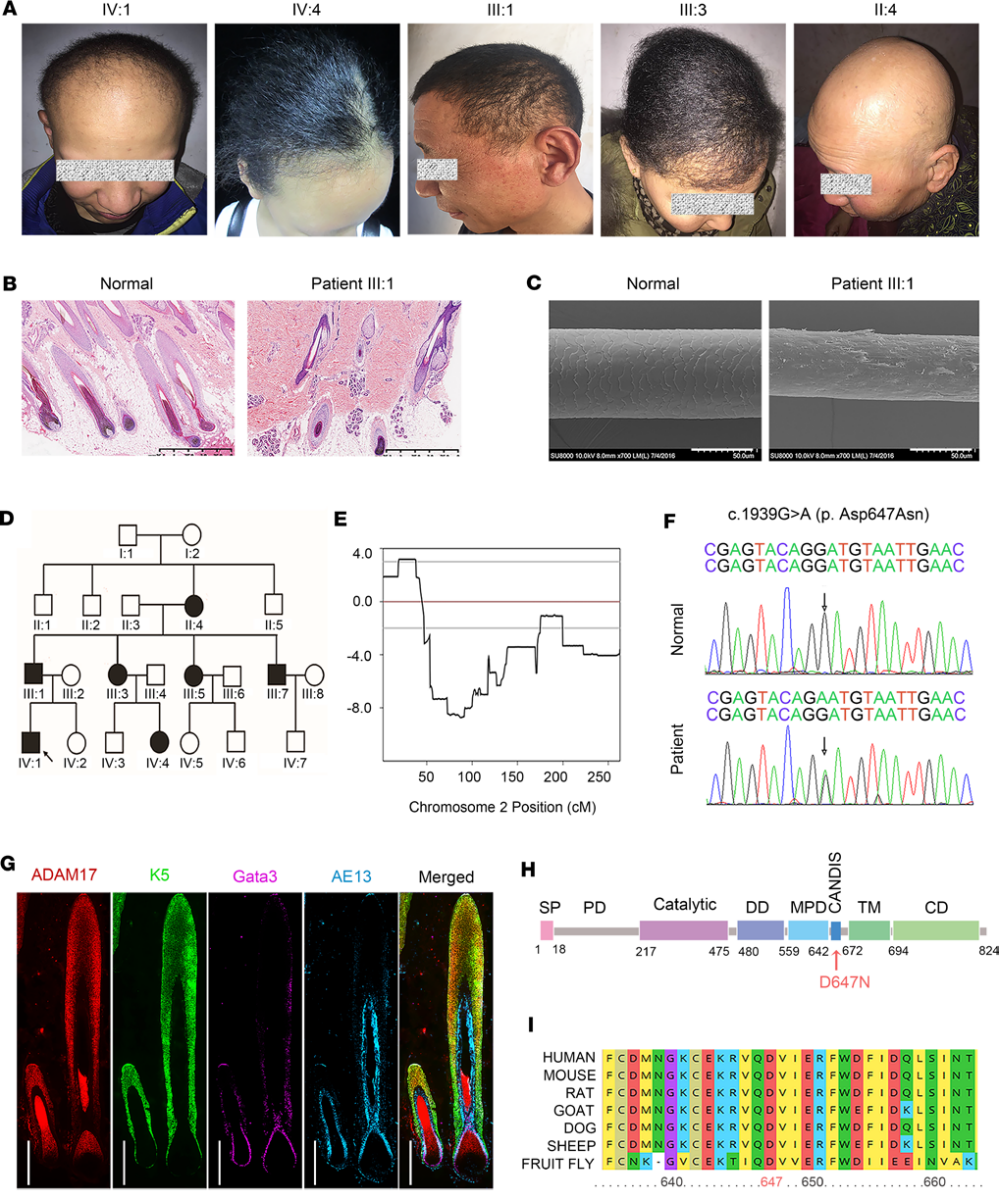
*ADAM17* variant leads to ADWH. (**A**) Representative clinical pictures of patients with ADWH. (**B**) Hematoxylin and eosin (HE) staining of scalp tissues from patients and the normal controls. Scale bars, 125 μm. (**C**) The scanning electron microscopy (SEM) analysis revealed that the patient’s hair shaft displayed a nonuniform and abnormal cross-sectional shape. Furthermore, there was a substantial peeling of hair cuticle, which normally functions as the hair shaft’s outermost protective layer. Scale bars, 50 μm. (**D**) Pedigree of the family for patients. Hollow boxes represented the healthy men, while solid boxes represented the affected men. Hollow circles represented the healthy women, while solid circles represented the affected women in the family tree. The proband was identified with a black arrow. (**E**) A genome-wide linkage analysis provided evidence of linkage to chromosome 2, with a maximum LOD score of 3.18. (**F**) Gene sequencing revealed the heterozygous *ADAM17* p.Asp647Asn (c.1939G>A) variant in patients. The arrows indicate the variant. (**G**) ADAM17 was predominantly expressed in the hair cortex, inner root sheath (IRS), and outer root sheath (ORS) of human hair follicle. Scale bar, 500 μm. (**H**) Schematic overview of ADAM17 protein and its domains. The p.D647N mutation was localized in CANDIS domain (indicated by a red arrow). SP, signal peptide; PD, pro-domain; Catalytic, catalytic metalloprotease domain; DD, disintegrin domain; MPD, membrane proximal domain; CANDIS, conserved ADAM17 seventeen dynamic interaction sequence; TM, transmembrane domain; CD, cytoplasmic domain. (**I**) Aspartic residue at position 647 is located within the conserved ADAM17 dynamic interaction sequence (CANDIS) domain of ADAM17, which is highly conserved.

We conducted a study on a Chinese family consisting of 7 patients with generalized hypotrichosis and curly hair. The majority of affected individuals exhibited tightly curled and sparse hair either at birth or during the early stage of infancy. The men exhibited varying degrees of hypotrichosis, often displaying fragile and thin scalp hair. Notably, they also had woolly hair and patchy hair loss of eyebrows, eyelashes, beard, mustache, axilla, and body. Hair growth tended to be limited, at a rate of 1–2 cm since birth throughout their lifetime, leading to complete hair loss as they aged. Conversely, women presented milder symptoms in comparison with men, often showing only woolly hair and punctate or flaky alopecia (Figure 1A). No abnormalities were detected concerning their skin, nails, teeth, or sweating. Hematoxylin and eosin (HE) staining of scalp sections exhibited a marked reduction in hair follicle counts, with minimal inflammatory infiltration observed around hair follicles (Figure 1B). Scanning electron microscopy (SEM) imaging of healthy individuals’ hair revealed a consistent ceramic tile–like arrangement of hair cuticles, whereas a patient’s hair displayed longitudinal grooves and cleavages. Moreover, the patient’s hair exhibited irregular transverse sections and widespread exfoliation of the hair cuticle (Figure 1C).

The provided pedigree offered persuasive evidence supporting the autosomal dominant mode of inheritance for the phenotypes (Figure 1D). To identify potential pathogenic variants associated with hypotrichosis causative genes like *KRT74*, *KRT25*, *KRT71*, *APCDD1*, *RPL21*, *SNRPE*, *CDSN*, *U2HR*, *EPS8L3*, *HR*, *DSG4*, *LIPH*, *LPAR6*, *DSC3*, *KRT25*, *LSS*, *TTMP*, *KRT86*, *KRT83*, and *KRT81*, gene panel sequencing was used. However, the variant search yielded no results. We performed a genome-wide linkage analysis approach using single nucleotide polymorphisms (SNPs) and found evidence for linkage to chromosome 2 with a maximum log of the likelihood odds ratio (LOD) score of 3.18 (Figure 1E). Subsequently, we narrowed down the candidate region to 19.6 cM, between markers rs979290 and rs57254657, which encompassed 126 annotated genes, through genotyping of markers in the identified region (Supplemental Figure 1; supplemental material available online with this article; https://doi.org/10.1172/jci.insight.177588DS1). We conducted whole-exome sequencing on an affected family member (II:4), screening all variants in the critical region, and detected a heterozygous missense mutation c.1939G>A (p.Asp647Asn) in the *ADAM17* gene (NM_003183). Upon conducting Sanger sequencing on all family members, we identified that the mutation was detected only in affected family members (Figure 1F). All identified variants were absent from public databases (1,000 Genomes, ClinVar, Ensembl, and gnomAD) and 600 healthy controls.

Furthermore, ADAM17 is predominantly expressed in the hair cortex, IRS, and outer root sheath (ORS) of hair follicle (Figure 1G and Supplemental Figure 2). Aspartic residue at position 647 is located within the CANDIS domain of ADAM17, which is highly conserved and plays a vital role in protein interactions (Figure 1, H and I).

### Adam17 (p.D647N) mutant mouse model mimics hair loss in HYPT.

**Figure 2:**
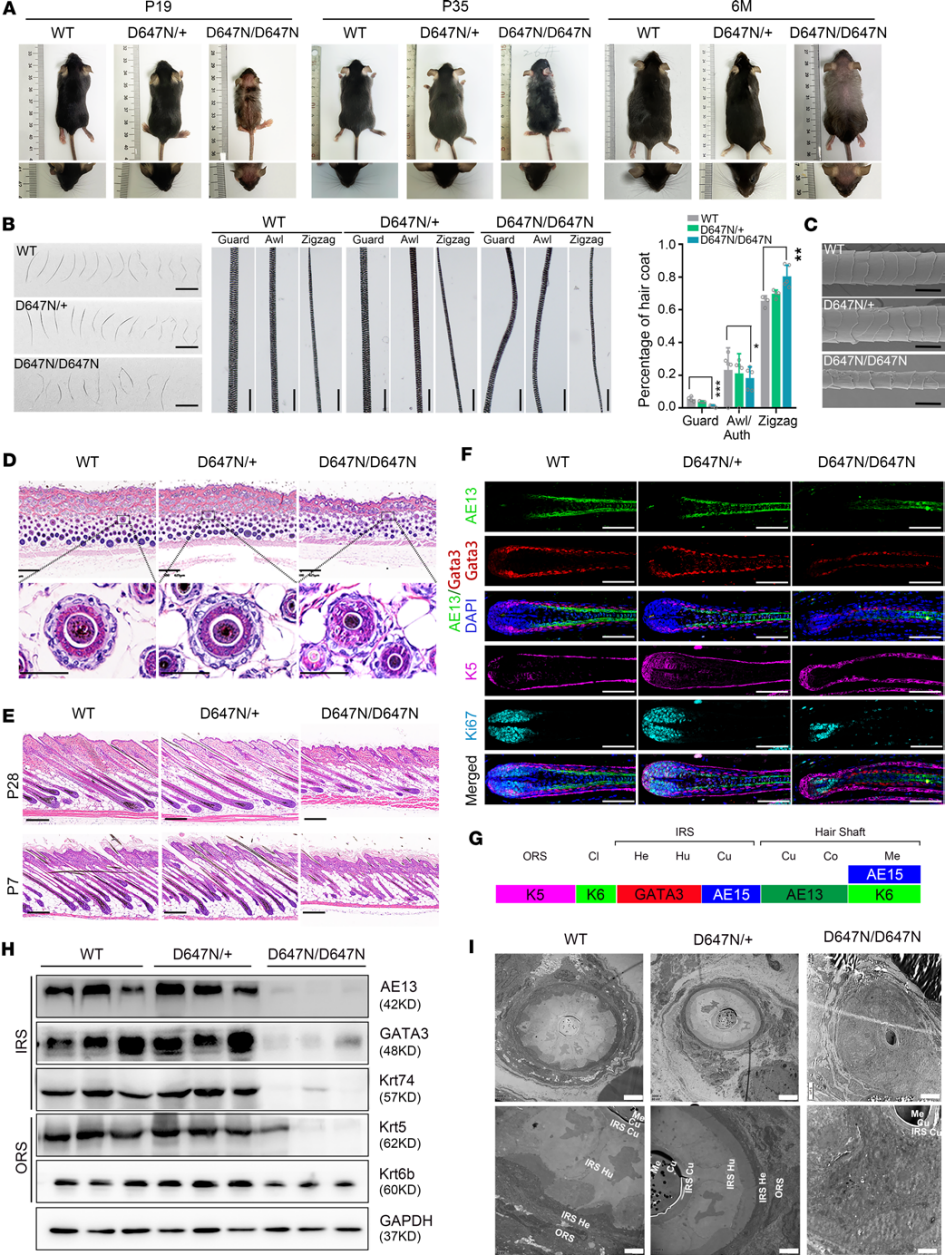
*Adam17* (p.D647N) variant causes abnormal hair follicle morphology and hair loss in mice. (**A**) Hair coats and vibrissa hairs of wild-type (WT), Adam17^D647N/+^, and Adam17^D647N/D647N^ mice at postnatal day (P) 19, P35, and 6 months (M). (**B**) Pelage hairs were observed under an optical microscope. Left and middle panel: compared with wild-type hairs, all 4 hair types of Adam17^D647N/D647N^ pelage hairs showed waviness. Scale bars, 2 mm. Right panel: Adam17^D647N/D647N^ mice displayed a significantly reduced proportion of primary and secondary hairs and a significant increase in the proportion of zigzag hairs. (*n* = 4 biological replicates.) (**C**) The scanning electron microscopy (SEM) analysis unveiled a peeling of the hair cuticle in the hair of Adam17^D647N/D647N^ mice. Scale bar, 10 μm. (**D**) HE staining revealed notable structural abnormalities of hair follicles (HFs) in Adam17^D647N/D647N^ mice at P28. Upper panel scale bars, 250 μm; lower panel scale bars, 50 μm. (**E**) HE staining of longitudinal sections revealed obvious structural abnormalities of HFs in Adam17^D647N/D647N^ mice during the first anagen (P7) and second anagen (P28). Scale bars, 250 μm. (**F**) Immunofluorescence staining revealed a substantial decrease in IRS markers within the HFs of Adam17^D647N/D647N^ mice, suggesting significant morphological abnormalities of the IRS. Scale bar, 100 μm. (**G**) A schematic illustration of HF layers and markers expressed in HF. (**H**) Immunoblot analysis of HF layer-specific markers in dorsal skin. (**I**) Transmission electron microscopy (TEM) of HFs at approximately 500 μm depth. Left panel scale bar, 5 μm; right panel scale bar, 2 μm. ORS, outer root sheath; IRS, inner root sheath; Cl, companion layer; He, Henle’s layer; Hu, Huxley’s layer; Cu, cuticle; Co, cortex; Me, medulla. All experiments were repeated 3 times. Data were expressed as mean ± SD; *P < 0.05; **P < 0.01; ***P < 0.001; 1-way ANOVA (**B**).

In order to ascertain whether the *ADAM17* (p.D647N) mutation is pathogenic, we constructed and *Adam17* (p.D647N) knockin mouse model using CRISPR/Cas9-mediated genome engineering (Supplemental Figure 3, A and B). Homozygous mice (Adam17^D647N/D647N^) were distinguishable from wild-type or heterozygous littermates (Adam17^D647N/+^) by their abnormal hair characteristics, including wavy vibrissa hair and a thinner, wavy, gray coat (Figure 2A). These distinctive features first appeared shortly after birth and gradually worsened as the mice aged (Figure 2A). Four hair follicle types with varying shapes and sizes emerged during hair development in the dorsal skin of mice, including guard (primary), auchene and awl (secondary), and zigzag (tertiary) hairs (28). Compared with the wild-type, Adam17^D647N/D647N^ pelages exhibited a reduction in primary and secondary hairs but a significant increase in the proportion of zigzag hairs. All 4 hair types in the Adam17^D647N/D647N^ pelage displayed waviness, disorganized medulla, and irregular melanin piles (Figure 2B). Furthermore, SEM revealed irregular and broken scales, suggesting a defect in the hair cuticle (Figure 2C).

HE staining showed that Adam17^D647N/D647N^ mice exhibited a diminished count of hair follicles and abnormal hair architecture at the first anagen (P7) and the second anagen (P28) (Figure 2, D and E). Hair follicles in wild-type mice exhibited distinctive concentric layers, namely ORS, companion layer, IRS (Henle’s layer, Huxley’s layer, and cuticle), and hair shaft (cuticle, cortex, and medulla). However, the concentric structure of hair follicles in Adam17^D647N/D647N^ mice was disrupted, along with a disturbed distribution of cells in the IRS (Figure 2D). Additionally, hair shafts in the Adam17^D647N/D647N^ mice displayed irregularities and deformities, suggesting a potential role for ADAM17 in hair shaft development (Figure 2E). Immunofluorescence demonstrated that IRS labeled by Gata3 and hair cortex keratins labeled by the AE13 antibody were markedly diminished in the hair follicles of Adam17^D647N/D647N^ mice. Conversely, there was a noticeably increased expression of K5, which functioned as a pivotal marker for ORS (Figure 2F). Furthermore, Ki67 immunostaining revealed a decreased cell proliferation ability in hair germ of Adam17^D647N/D647N^ mice (Figure 2F). These observations were supported by Western blotting (Figure 2, G and H, and Supplemental Figure 3C). In addition, transmission electron microscopy (TEM) demonstrated that hair follicles in wild-type mice exhibited distinctive concentric layers, while all layers apart from the hair shaft and IRS cuticle of hair follicles in Adam17^D647N/D647N^ mice were indistinguishable (Figure 2I). Overall, these results suggested that the p.D647N mutation in *ADAM17* had a substantial impact on the proper organization of hair follicles.

During the weaning period, Adam17^D647N/D647N^ mice exhibited a distinguishable shiny, red, and semitransparent skin phenotype. Subsequently, at P15, a notable incidence of erythroderma developed along with a substantial amount of desquamation (Supplemental Figure 3D). Ultimately, this affliction resolved spontaneously in the subsequent days. Additionally, Adam17^D647N/D647N^ mice experienced notable developmental retardation within the first 4 months (Supplemental Figure 3E). Remarkably, these mice displayed edema in their extremities and a significantly high mortality rate during the peri-weaning period (Supplemental Figure 3F). Conversely, no discernible anomalies were detected in the fur or skin of Adam17^D647N/+^ mice as per our observation (Figure 2 and Supplemental Figure 3).

### Disruption of HFSCs in Adam17-mutant mice contributes to the woolly hair and alopecia phenotype.

**Figure 3:**
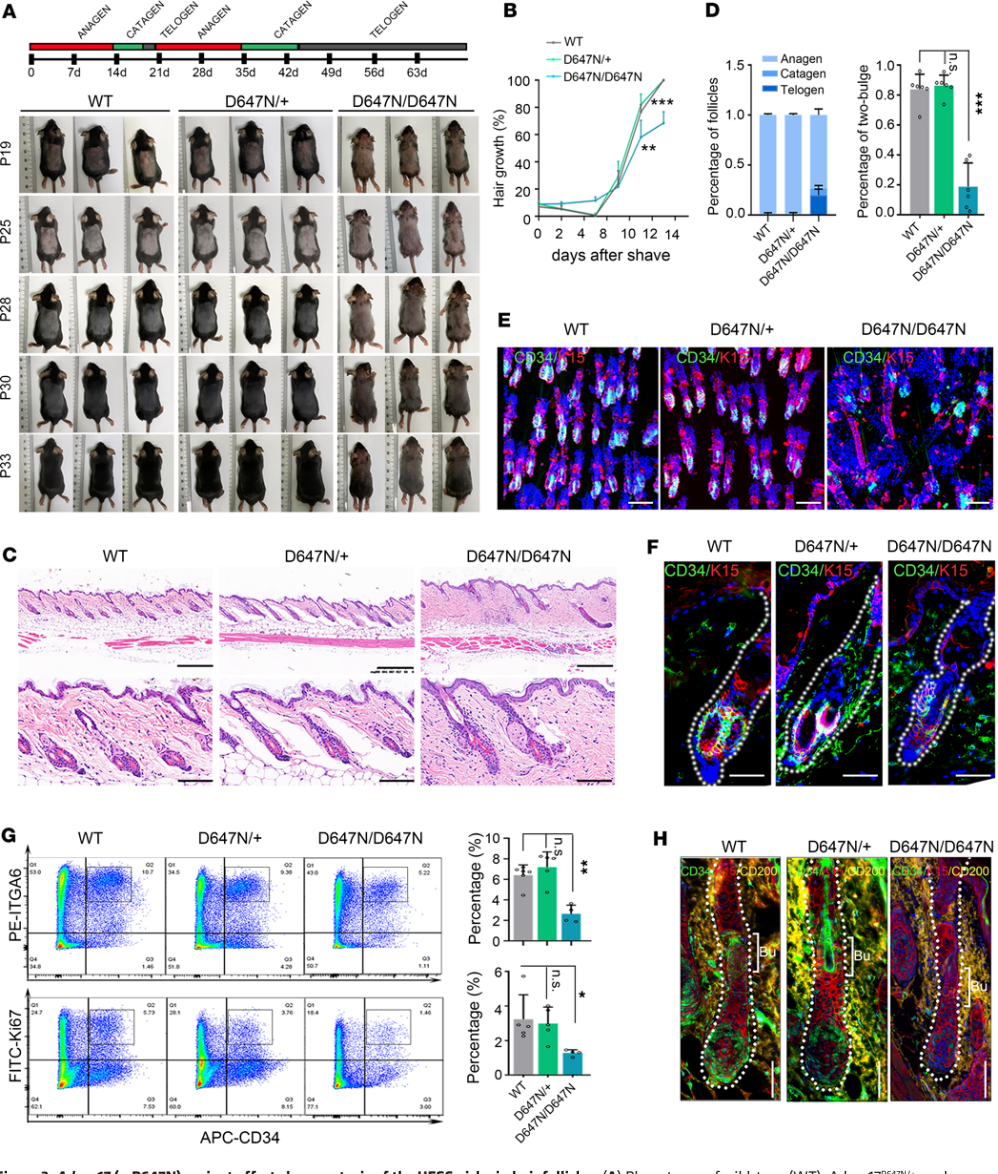
*Adam17* (p.D647N) variant affects homeostasis of the HFSC niche in hair follicles. (**A**) Phenotypes of wild-type (WT), Adam17^D647N/+^, and Adam17^D647N/D647N^ mice after shaving back skin during telogen (P19) and monitoring initiation of the next hair cycle. *Adam17* mutation impeded hair regeneration. (**B**) Statistical data on the proportion of the skin with pigmentation in mice after hair shaving. (*n* = 6 biological replicates.) (**C**) HE staining of back skin during second telogen (P63). Upper panel scale bars, 300 μm; lower panel scale bars, 50 μm. (**D**–**F**) WT hair follicles (HFs) possessed a 2-bulge architecture, whereas Adam17^D647N/D647N^ HFs usually had only 1. (**D**) Statistical data on the proportion of 3 HF types. (*n* = 6 biological replicates.) (**E**) Immunofluorescence was performed on whole-mount back skin hair follicles at the second telogen stage using HFSC markers K15 and CD34. Scale bar, 100 μm. (**F**) Skin sections underwent immunofluorescence staining using antibodies specific to HFSC markers. Scale bars, 40 μm. (**G**) Fluorescence-activated cell sorting (FACS) analyses of HFSC populations sorted by high α6-integrin and CD34. Right upper panel: Quantification of CD34-positive/α6-high cells (indicated by the black square brackets) among epithelial cells in second telogen mice. (*n* = 4–6 biological replicates.) Right lower panel: Quantification of CD34-positive/Ki67-positive cells among epithelial cells in second telogen mice. (*n* = 4–6 biological replicates.) (**H**) HFSC differentiation was blocked in Adam17^D647N/D647N^ mice. Scale bars, 40 μm. CD200, the marker of secondary hair germ. Bu, hair bulge. All experiments were repeated 3 times. Data were expressed as mean ± SD; *P < 0.05; **P < 0.01; ***P < 0.001; 1-way ANOVA (**B** and **D**); Mann-Whitney test (**G**: right upper panel); Kruskal-Wallis test (**G**: right lower panel).

To further explore whether HFs in Adam17^D647N/D647N^ mice are defective in forming new hair, we shaved hair and monitored the appearance of skin pigmentation. Adam17^D647N/D647N^ mice exhibited a delayed onset of anagen in a partial area of dorsal skin compared with their wild-type counterparts (Figure 3, A and B). This observation suggested that the *Adam17* (p.D647N) mutation hindered hair regeneration. HFs generate a new bulge beside the old one and persist to the next cycle in the telogen phase, which is called club hair (29). HE staining demonstrated a remarkable decrease of 2-bulge HFs in telogen phase (P63) (Figure 3, C and D). CD34 and K15 are specifically expressed in the inner and outer layers of the club hair, respectively, and are commonly used to label HFSCs (30). The whole-mount immunostaining against CD34 and K15 revealed that wild-type HFs developed a 2-bulge architecture, whereas Adam17^D647N/D647N^ mice usually had only 1 (Figure 3D, right panel, and Figure 3, E and F).

At telogen, both wild-type and mutant bulge cells consisted of an α6 integrin–rich (ITGA6-rich) basal layer and an ITGA6-low suprabasal layer, both of which were CD34^+^. Adam17^D647N/D647N^ follicles displayed fewer CD34^+^ bulge cells by fluorescence-activated cell sorting (FACS) than wild-type follicles (Figure 3G). Adam17^D647N/D647N^ follicles displayed lower Ki67 positivity than wild-type follicles (Figure 3G). Immunofluorescence of CD200, the marker of secondary hair germ, verified a decreased differentiation of HFSCs in Adam17^D647N/D647N^ mice at early stage of anagen (P21) (Figure 3H). Taken together, we find that the *Adam17* (p.D647N) mutation leads to HFSC abnormalities.

### ADAM17 mutation decreases its protein stability owing to enhanced auto-ubiquitination.

**Figure 4:**
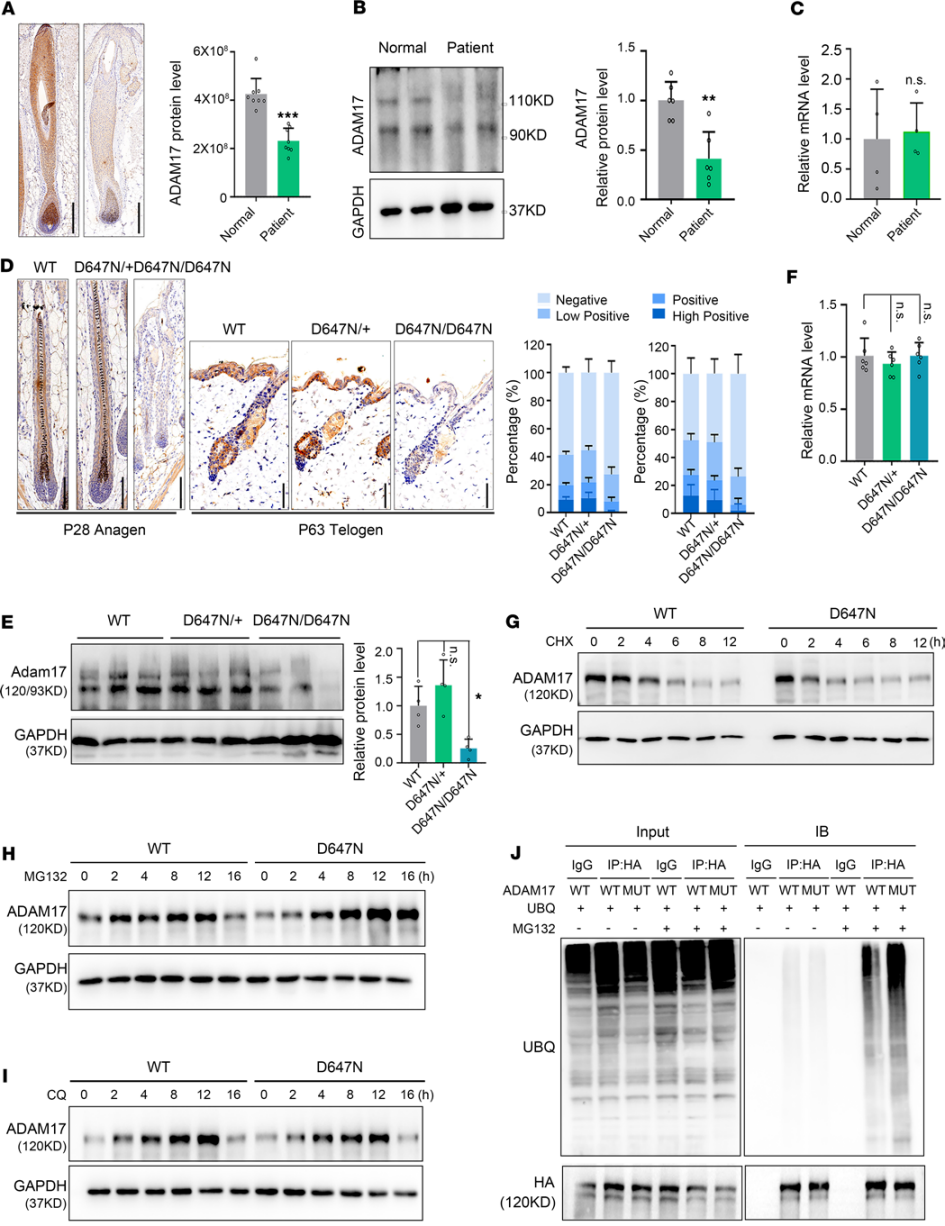
*ADAM17* (p.D647N) variant decreases its protein stability owing to enhanced auto-ubiquitination. (**A**) Immunohistochemical staining showed that the expression of ADAM17 in patients’ hair follicles (HFs) was significantly lower than that in normal controls. Scale bar, 300 μm (*n* = 8 biological replicates of normal controls; *n* = 8 technical replicates of patient III:1). (**B**) ADAM17 protein level in the scalp tissues of patients was lower than that in the controls. (*n* = 6 biological replicates of controls; *n* = 6 technical replicates of patient III:1.) (**C**) No significant alteration detected in the mRNA levels of *ADAM17*. (*n* = 4 biological replicates of controls; *n* = 4 technical replicates of patient III:1.) (**D**) Adam17 protein level in HFs of Adam17^D647N/D674N^ mice was lower than that in wild-type (WT) mice. Left scale bar, 100 μm; right scale bar, 50 μm. (*n* = 28–32 technical replicates.) (**E**) Adam17 protein level in skin tissues was significantly reduced in Adam17^D647N/D674N^ mice compared with the WT mice. (*n* = 3 biological replicates.) (**F**) No significant changes were observed in the mRNA levels of *Adam17* between ADAM17^D647N/D674N^ and WT mice. (*n* = 6 biological replicates.) (**G**) Cycloheximide (CHX) chase analysis showed that *ADAM17* (p.D674N) mutation induced rapid degradation of ADAM17 in HaCaT cells. (**H**) *ADAM17* (p.D647N) mutation resulted in heightened degradation of ADAM17 though proteasome pathway in HaCaT cells. (**I**) *ADAM17* (p.D647N) mutation had no bearing on the degradation of ADAM17 through the autophagy pathway in HaCaT cells. (**J**) *ADAM17* (p.D647N) mutation resulted in heightened ubiquitination and subsequent degradation of ADAM17 via the proteasomal pathway. All experiments were repeated 3 times. Results were expressed as mean ± SD; *P < 0.05; **P < 0.01; ***P < 0.001; unpaired 2-tailed *t* test (**B** and **C**); 1-way ANOVA (**E** and **F**); Mann-Whitney test (**A**).

To elucidate the potential impact of the p.D647N mutation on ADAM17, we analyzed its expression in humans and mouse samples. Notably, our investigations revealed a significant reduction of ADAM17 protein levels in affected individuals compared with healthy controls (Figure 4, A and B), though we did not observe any significant differences in *ADAM17* mRNA levels (Figure 4C). Correspondingly, we observed a decrease in the protein level of Adam17, instead of mRNA levels, in hair follicles of Adam17^D647N/D647N^ mice (Figure 4, D–F). We postulated that this might be attributable to the differential stability of 2 proteins, as mRNA levels could not account for the difference in protein levels. To investigate the stabilities of 2 proteins, we stably overexpressed wild-type and mutant ADAM17 in HaCaT cells, an immortalized human keratinocyte line. Cycloheximide chase assays results indicated that the mutant ADAM17 displayed a shorter half-life compared with the wild-type counterpart (Figure 4G and Supplemental Figure 4A). Subsequently, we treated HaCaT cells with the proteasome inhibitor MG132 or lysosome inhibitor chloroquine (CQ). Our findings revealed that MG132, but not CQ, deterred the reduction of the mutant ADAM17 protein level relative to the wild-type counterpart, suggesting that the ubiquitin/proteasome pathway was involved in the further degradation of the mutant ADAM17 expression (Figure 4, H and I, and Supplemental Figure 4, C and E). Additionally, we verified that *Adam17* (p.D647N) mutation decreased its protein stability via ubiquitin/proteasome pathway in primary cultured fibroblasts from wild-type and Adam17^D647N/D647N^ mice (Supplemental Figure 4, B, D, and F). Indeed, hyperubiquitination of mutant ADAM17 was observed in HEK293T cells (Figure 4J). In summary, our findings indicate that the mutant ADAM17 displays elevated auto-ubiquitination levels, ultimately leading to decreased protein stability and lower abundance.

Furthermore, we evaluated the shedding activity of ADAM17 in human skin tissue, as well as in primary cultured mouse skin fibroblast cells and HaCaT cells. Our findings indicated a significant reduction in ADAM17 shedding activity in the skin tissue of patients and primary cultured mouse skin fibroblast cells (Supplemental Figure 5, A and B). This reduction may be attributed to a decrease in the expression of ADAM17 (Figure 4, A–C, and Supplemental Figure 4B). It is worth noting that we overexpressed wild-type and mutant ADAM17 in HaCaT cells and observed no significant difference in shedding activity between the 2 groups at identical ADAM17 expression levels (Supplemental Figure 5C). These results suggest that *ADAM17* variant does not impact its shedding activity.

### ADAM17 mutation reinforces the bond between ADAM17 and its specific E3 ubiquitin ligase, TRIM47.

**Figure 5:**
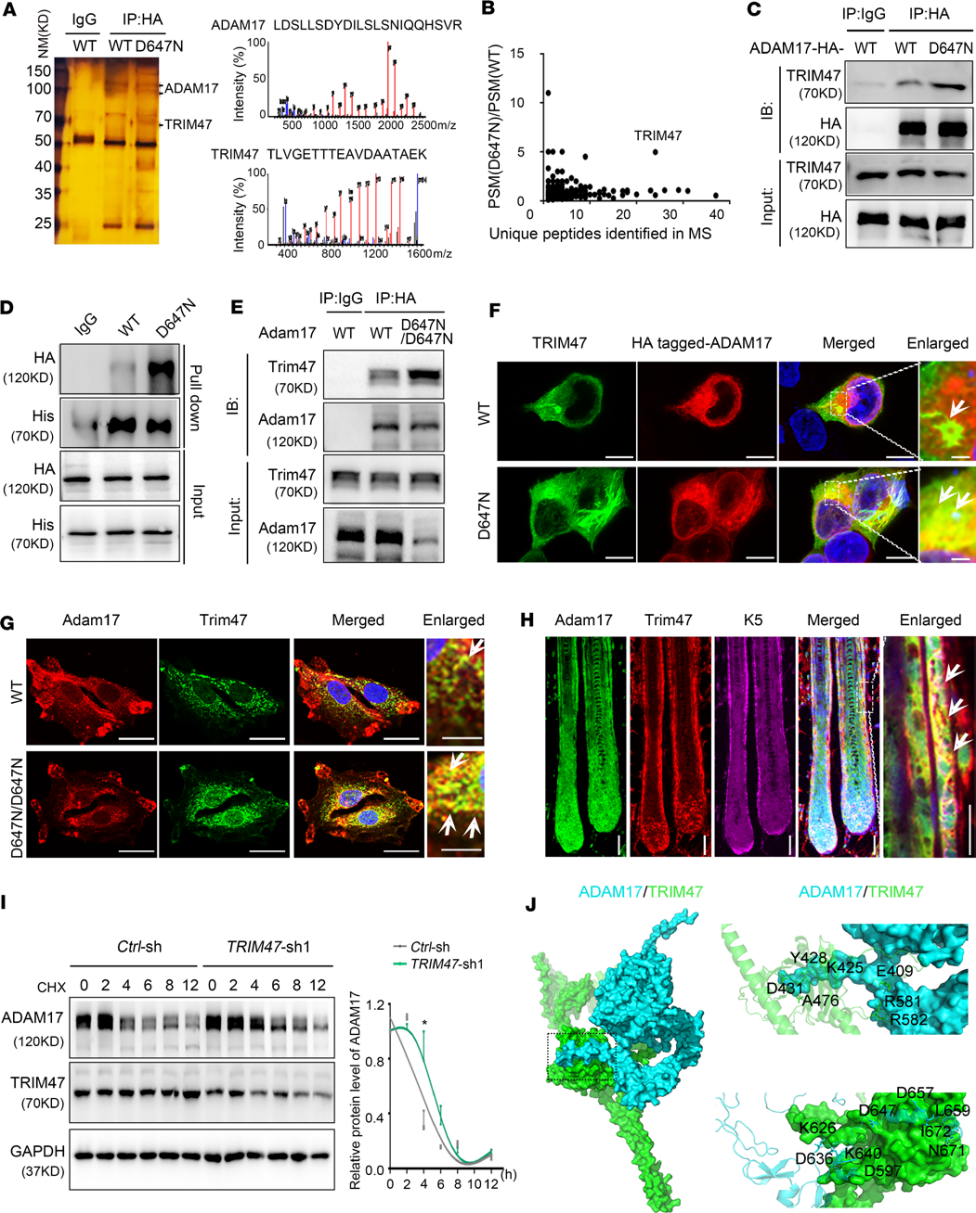
TRIM47 is identified as a specific E3 ubiquitin ligase of ADAM17. (**A**) Liquid chromatography-tandem mass spectrometry (LC-MS/MS) analysis of wild-type and mutant ADAM17 binding proteins. (**B**) Peptide spectrum match score ratios for the proteins identified by mass spectrometry. (**C**) ADAM17 interacts with TRIM47 in HaCaT cells, and their association was enhanced by the *ADAM17* (p.D647N) mutation. (**D**) The direct interaction between ADAM17 and TRIM47 was validated by pulldown assay. (**E**) Endogenous Adam17 binds to Trim47 in the epidermal tissue lysates obtained from mice, and *Adam17* (p.D647N) mutation enhances their association. (**F**) Confocal immunofluorescence revealed a colocalization (yellow) of ADAM17 (red) and TRIM47 (green) in HaCaT cells, and *ADAM17* (p.D647N) mutation enhanced their colocalization. Scale bars, 8 μm (first 3 images); 1 μm (fourth image). (**G**) Confocal immunofluorescence revealed a colocalization (yellow) of Adam17 (red) and Trim47 (green) in primary cultured mouse skin fibroblasts. Scale bars, 10 μm (first 3 images); 1 μm (fourth image). (**H**) Adam17 and Trim47 colocalized in both IRS and ORS of hair follicles. The white arrows indicated the colocalization (yellow) of Trim47 (green) and Adam17 (red). Scale bars, 40 μm (first 4 images); 20 μm (fifth image). (**I**) Knockdown of *TRIM47* impeded the proteasomal degradation of ADAM17. Left panel: Representative immunoblot images of the ADAM17 and TRIM47 protein levels during CHX chase assays. Right panel: quantification of immunoblotting results corresponding to the left panel. (*n* = 3 biological replicates.) (**J**) The 3-dimensional structure of ADAM17/TRIM47 complex in stereo. Right upper panel: essential amino acids of ADAM17 (blue) that polar contacted TRIM47 (green) were depicted. Right lower panel: residues of TRIM47 (green) which polar contacted to ADAM17 (blue) were depicted. All experiments were repeated 3 times. Results were expressed as mean ± SD; *P < 0.05; unpaired 2-tailed *t* test (**I**).

E3 ubiquitin ligases exhibit substrate specificity by selectively binding to target proteins, which results in their ubiquitination and subsequent degradation. To gain insight into the molecular mechanisms underlying *ADAM17* (p.D647N) mutation causing reduced protein stability, we set out to identify its E3 ubiquitin ligase by co-immunoprecipitation (co-IP) coupled with mass spectrometry. Through in-depth bioinformatic analysis of potential ADAM17-binding proteins identified, we located RING-E3 ligase TRIM47 as a potential interactor of ADAM17 (Figure 5, A and B, and Supplemental Table 1). We validated the direct interaction between ADAM17 and TRIM47 using co-IP and pulldown assay (Figure 5, C and D). The endogenous association between Adam17 and Trim47 was then verified by co-IP assay in the epidermal tissue lysates of mice (Figure 5E). Confocal immunofluorescence revealed that Adam17 and Trim47 were colocalized in HaCaT cells, primary cultured mouse skin fibroblasts, and HFs of mice (Figure 5, F–H). Moreover, TRIM47 showed high expression in HFSCs (Supplemental Figure 6A). *ADAM17* (p.D647N) mutation strengthened the association between ADAM17 and TRIM47, verifying the mass spectrometry results (Figure 5, B–G). Subsequent to the aforementioned validation of direct interaction between ADAM17 and TRIM47, we probed the effect of knockdown/overexpression of TRIM47 in mutant ADAM17 HaCaT cells (Supplemental Figure 6B). Our results indicated that knockdown of TRIM47 impaired the degradation of the mutant ADAM17 (Figure 5I), while overexpression of TRIM47 accentuated degradation of the mutant ADAM17 (Supplemental Figure 6C). These results indicate that *ADAM17* (p.D647N) mutation reinforces the bond between ADAM17 and TRIM47, ultimately resulting in a reduced protein stability.

A computational 3D complex structural model by ZDock based on the x-ray crystal structure from the Protein Data Bank was further generated (Figure 5J, left panel). Docking simulation data from the model demonstrated that amino acids D597, K626, D636, K640, D647, D657, L659, N671, and I672 of ADAM17 form a “hairpin” structure (Figure 5J, right upper panel) and that amino acids F409, K425, Y428, D431, A476, R581, and R582 of TRIM47 form a “hairpin” structure (Figure 5J, right lower panel). Based on the 3D complex structure model, it was deduced that these hairpin-like structures were accountable for facilitating their interaction.

### ADAM17 deficiency blocks the Notch signaling pathway, consequently impairing HF development.

**Figure 6:**
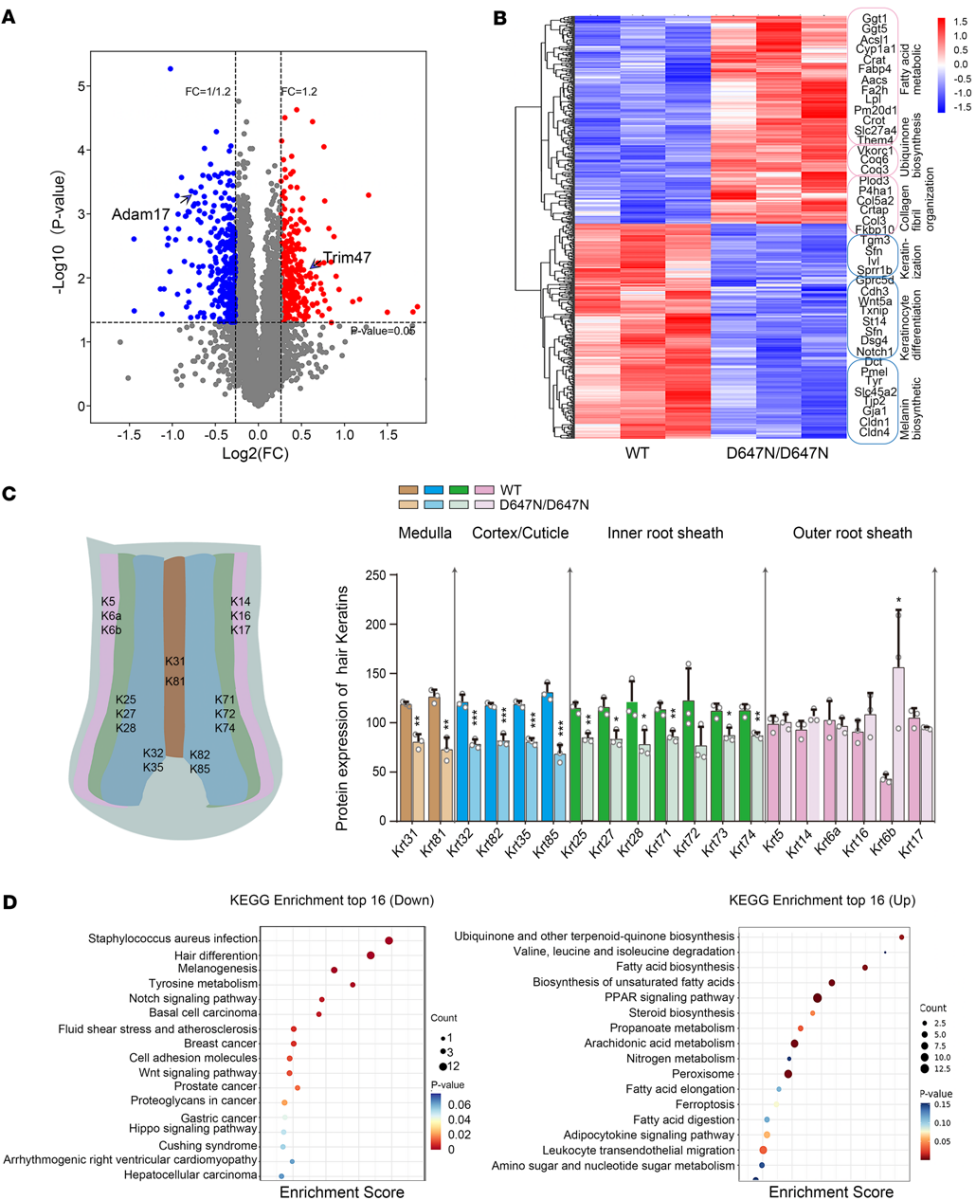
ADAM17 variant inhibits hair follicle development through the Notch signaling pathway. (**A**) Log_2_ fold-change in the normalized counts of Proteomics-Seq reads of differentially expressed proteins (DEPs) in wild-type and Adam17^D647N/D647N^ mice. (**B**) Heatmap of DEPs in the indicated mice, annotated for selected proteins. (**C**) DEP analysis revealed a substantial decrease of hair shaft and IRS markers in Adam17^D647N/D647N^ mice. (*n* = 3 biological replicates.) (**D**) Pathway enrichment analysis of DEPs revealed marked upregulation of ubiquinone and other terpenoid-quinone biosynthesis pathways and downregulation of Notch signaling pathways. Results were expressed as mean ± SD; *P < 0.05; **P < 0.01; ***P < 0.001; unpaired 2-tailed *t* test (**C**).

To explore intrinsic effects resulting from the loss of ADAM17 expression, we performed proteomic analysis on the dorsal skin during the anagen phase (P28). Analysis of differentially expressed proteins (DEPs) revealed that *Adam17* variant led to broad changes in protein levels (Figure 6, A and B). Specifically, we detected a significant increase in ubiquinone and other terpenoid-quinone biosynthesis enzymes (Vkorc1, Coq6, and Coq3), along with the upregulation of the Adam17-specific E3 ubiquitin ligase Trim47 (Figure 6, A and B, and Supplemental Figure 6D). Then we verified the upregulation of TRIM47 in mouse skin samples and HaCaT cells (Supplemental Figure 6, E–G). Our study revealed that *Adam17* mutation markedly affected the proper development and organization of HFs. Indeed, significant reductions were observed in proteins related to keratinocyte differentiation and HF morphogenesis (Figure 6B), especially proteins linked to hair shaft and IRS structure (Figure 6C). Furthermore, Kyoto Encyclopedia of Genes and Genomes (KEGG) pathway enrichment analysis demonstrated decreased activity within structural homeostasis-related pathways, including Notch signaling pathway and cell adhesion pathway (Figure 6D).

**Figure 7:**
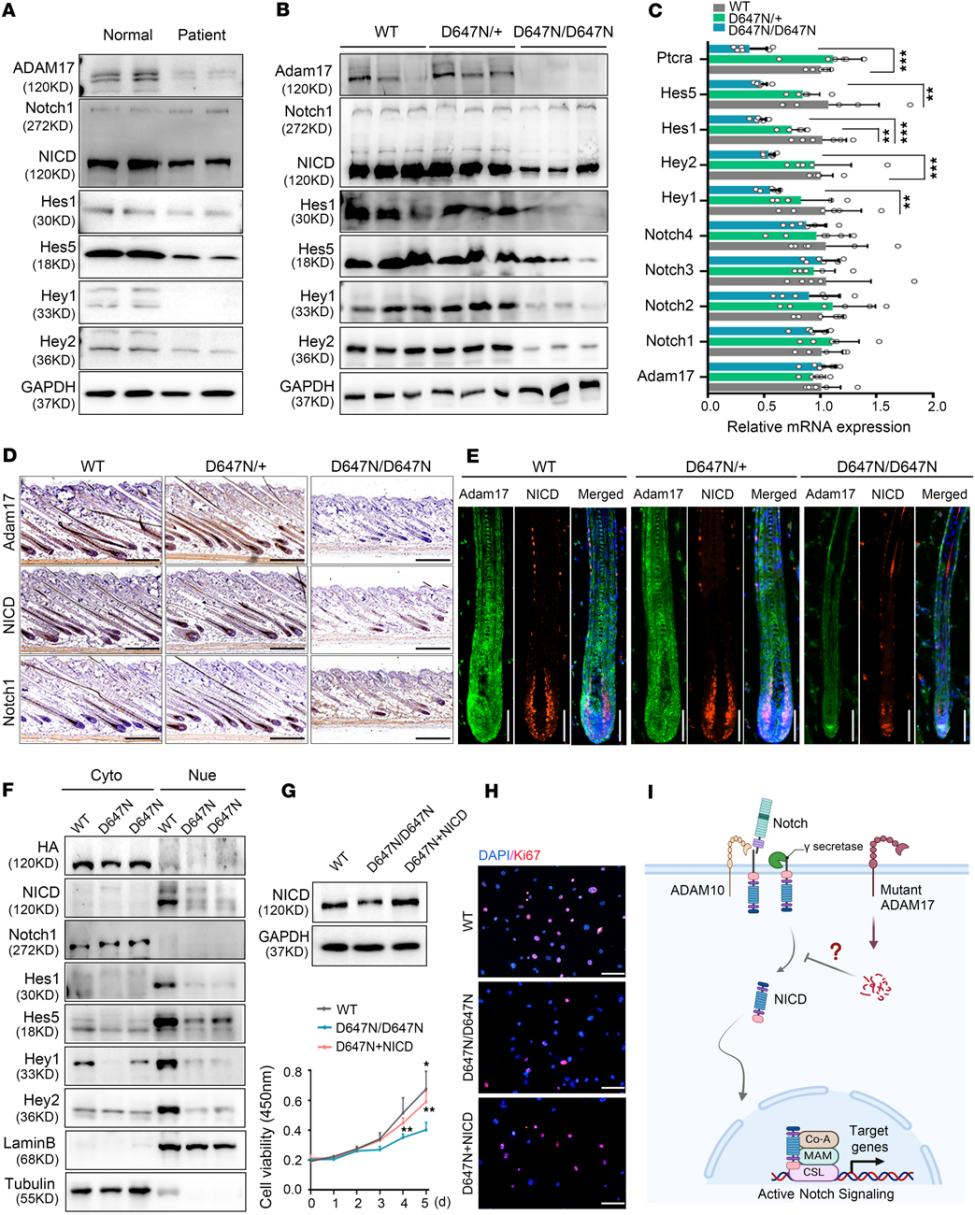
*ADAM17* (p.D647N) variant affects Notch signaling, resulting in hair follicle malformation. (**A**) Effects of *ADAM17* mutation on protein levels of key molecules involved in Notch signaling observed in the skin biopsy of the patient. (**B**) *Adam17* mutation inhibited Notch signaling in the skin tissues of mice. (**C**) Effects of *Adam17* mutation on mRNA levels of key molecules involved in Notch signaling observed in the skin tissues of mice. (*n* = 6 biological replicates.) (**D**) Immunohistochemical staining showed that *Adam17* mutation led to downregulation of ADAM17 and Notch intracellular domain (NICD) expression but not full-length Notch1 in Adam17^D647N/D647N^ mice. Scale bar, 250 μm. (**E**) Immunofluorescence showed that *Adam17* mutation led to downregulation of ADAM17 and NICD expression in hair follicle of Adam17^D647N/D647N^ mice. Scale bar, 80 μm. (**F**) Cellular component separation assay showed that NICD protein level was decreased in nucleus of ADAM17-mutant HaCaT cells. Lamin B2 and α-tubulin were used as the nuclear (Nue) and cytosolic (Cyto) protein makers, respectively. (**G** and **H**) Overexpressing NICD significantly rescued the proliferation activity of primary fibroblasts derived from Adam17^D647N/D647N^ mice. (*n* = 3 biological replicates.) (**I**) Schematic diagram of Notch signaling pathway. Following sequentially cleaving by ADAM10 and γ-secretase, Notch released its NICD. NICD then translocated to nucleus, and, in collaboration with RBPJ and Mastermind, activated the transcription of target genes, including members of the *Hes* and *Hey* families. All experiments were repeated 3 times. Results were expressed as mean ± SD; *P < 0.05; **P < 0.01; ***P < 0.001; 1-way ANOVA and Kruskal-Wallis test (**C**); Brown-Forsythe and Welch ANOVA tests (**G**).

Previous research has indicated that ADAM17 can shed TGF-α, which in turn actives EGFR signaling in HFs (18, 19). Thus, we conducted immunohistochemical staining to examine the influence of *Adam17* variant on the EGFR signaling pathway. Our findings revealed that the *Adam17* variant did not impact the expression of EGFR, as well as the phosphorylation of EGFR at tyrosine 992 (p-EGFR [Tyr992]) and tyrosine 1068 (p-EGFR [Tyr1068]) in Adam17^D647N/D647N^ mice (Supplemental Figure 7). Given that Notch signaling exerts regulatory effects on the differentiation of HFSCs into specific HF cell types, is it plausible that Notch is the underlying cause of the hair loss phenotype observed in ADAM17-mutant mice? The activity of key molecules in the Notch signaling pathway was investigated in humans and mouse samples. Ligand-mediated activation induces proteolytic cleavages of Notch and releases the Notch intracellular domain (NICD), which enters the nucleus and stimulates transcription of target genes (31). Our findings indicate a significant decrease in the protein level of NICD upon *ADAM17* variant, leading to decreased expression of Notch target genes, including *Hes1*, *Hes5*, *Hey1*, and *Hey2* (Figure 7, A and B, and Supplemental Figure 8, A and B). Real-time PCR (RT-PCR) analysis demonstrated that the *Adam17* mutation resulted in inhibited Notch target gene transcripts in mouse skin (Figure 7C). Immunohistochemistry and immunofluorescence assays revealed a reduced amount of NICD in the HFs of mice during anagen (Figure 7, D and E, and Supplemental Figure 8C). The nuclear and cytoplasmic protein extraction assays further verified that the *ADAM17* (p.D647N) variants effectively inhibited the Notch signaling pathway in keratinocytes. This is supported by the observed reduction in the expression of NICD and its target genes within the cell nucleus (Figure 7F). To further investigate the involvement of the Notch signaling pathway in *Adam17* mutation–induced HF malformation, we overexpressed NICD in primary cultured Adam17^D647N/D647N^ mouse skin fibroblasts. Our findings indicated that *Adam17* mutation significantly reduced the proliferation ability of primary fibroblasts, which could be rescued by overexpression of NICD (Figure 7, G and H, and Supplemental Figure 8, D and E). Collectively, our results validate the relationship between the *ADAM17* (p.D647N) mutation and Notch activation in HF development (Figure 7I).

## Discussion

This study presented compelling evidence for the involvement of ADAM17 in the growth of HFs and differentiation of HFSCs, underscoring its significance as a potent molecule in the development of HYPT. Our investigation revealed that *ADAM17* (p.D647N) variant caused HYPT in humans, and a *Adam17* (p.D647N) mutation mouse model successfully emulated sparse and woolly hair observed in HYPT. Moreover, detailed mechanistic investigations demonstrated that *ADAM17* mutation enhanced the interaction between ADAM17 and its E3 ubiquitin ligase, TRIM47, leading to increased auto-ubiquitination and consequent degradation of ADAM17. Then, reduced ADAM17 expression led to a decrease in the Notch signaling pathway, ultimately inhibiting the development of HFs.

Inherited hair loss disorders exhibit clinical and genetic heterogeneity, making it challenging to genotype patients accurately for diagnosis confirmation and genetic counseling. To date, over 14 detailed pathogenic genes of HYPT are reported to link with nonsyndromic hypotrichosis, including hereditary hypotrichosis simplex (HHS), Marie Unna hereditary hypotrichosis, localized autosomal recessive hypotrichosis (LAH), ADWH, and ARWH (6–8, 32–37) (Supplemental Table 2). HYPT3 and HYPT13 with pathogenic *KRT74* and *KRT71* genes, respectively, were also classified as ADWH subtypes 1 and 2 (7, 8). In this study, we identified a subtype of congenital hypotrichosis with woolly hair that we designated ADWH3, caused by a heterozygous mutation in *ADAM17*. Affected women exhibited curly hair resembling steel wool, while men displayed a range of phenotypic severity from limited to complete baldness, notwithstanding the pervasive thinning of body hair. Unlike HHS and LAH, which only affects scalp hair or is limited to some areas (36), ADWH3’s phenotypes were distinguished by a general scarcity of hair throughout the body. Nonetheless, typical of other forms of HYPT, progressive hair loss with age was also extensive (38, 39). Given the challenges associated with distinguishing this type of disorder from other congenital HYPT, genetic testing assumes critical importance.

Although the dominant variant in the *ADAM17* gene causes hypotrichosis with woolly hair in humans, homozygous variants are necessary to observe the phenotype in mice. Notably, the homozygous mice, exhibiting a more severe phenotype than heterozygous patients, were characterized by developmental retardation, edema in their extremities, and a markedly high mortality rate during the peri-weaning period. This phenotypic difference is likely due to genetic and biological disparities between mice and humans (40). It is important to note the phenotypes observed in animal models may vary from those observed in humans (41). For instance, HPRT deficiency does not constitute a pathological variant in mice, despite its association with severe Lesch-Nyhan syndrome in human hemizygotes (42, 43). In our study, we found that the heterozygous variant in the *ADAM17* gene impaired stability and exhibited haploinsufficiency in humans, while heterozygous variants maintained adequate protein levels, potentially averting hair development abnormalities in mice. However, further experimental investigation is necessary to fully understand the underlying mechanism.

ADAM17, a type I transmembrane protein, is composed of multiple domains. Mutations within these domains can lead to a range of disorders, indicating the sensitivity and delicacy of its structure and function. For instance, mutations in the PD domain have been identified as a key driver of colorectal cancer (44) and late-onset Alzheimer’s disease (10). Compound heterozygous mutations in the MPD and homozygous deletions in disintegrin domain result in neonatal inflammatory skin and bowel disease 1 (OMIM#614328) (45, 46). Notably, to date, there are no reported mutations in the highly conserved CANDIS domain. Here, we identified a heterozygous variant in the CANDIS domain of ADAM17 that caused nonsyndromic ADWH3. Further mechanistic investigations revealed that the variant in CANDIS enhanced ADAM17 susceptibility to ubiquitin-mediated degradation by enhancing its association with E3 ubiquitin ligase TRIM47. Ubiquitination is a critical posttranslational modification that plays a pivotal role in protein degradation via the proteasome (25, 47). Additionally, various posttranslational modifications play a crucial role in the rapid and reversible activation of ADAM17 (48–50). For instance, cytoplasmic phosphorylation of ADAM17 can regulate its activation, as well as rapid transport to the cell surface (51), which provides a mechanism for fine-tuning ADAM17 activity in response to cellular signals. Our studies enriched the understanding of the complex regulatory mechanisms that govern ADAM17 function and highlight the importance of structural conservation within the CANDIS domain.

Notch pathway is a highly conserved pathway, which exerts regulatory effects on the differentiation of HFSCs into specific HF cell types and represses their differentiation toward the epidermal cell fate (52). Notch signaling also regulates the skin microbiome and inflammation of the HFSC niche, protecting the HF from inflammatory damage (53). Notch1-deficient mice exhibited a thinner, shorter, and wavy appearance, accompanied by a defect in the hair cuticle (54), which were also observed in Adam17^D647N/D647N^ mice. In addition, we identified a marked decrease in the expression of key molecules associated with the Notch signaling pathway in both patients and Adam17^D647N/D647N^ mice. These findings establish a correlation between ADAM17 and Notch signaling, which has been previously reported in various diseases, including prostate tumors, non–small cell lung carcinoma, hepatocellular carcinoma, and diabetic nephropathy (55–57).

However, the classical viewpoint suggests ADAM10 directly cleaves the S2 site of Notch1, thereby releasing the NICD. Subsequently, the NICD translocates to the nucleus, where it interacts with the DNA-binding protein RBPJ and the coactivator Mastermind to facilitate the transcription of target genes, such as *Hes1* and *Hey1* (53, 58–61). Conversely, ADAM17 can only activate Notch signaling under nonphysiological conditions in vitro (62–66). ADAM10/Notch signaling axis–mediated regulation of host-microbe symbiosis crucially protects HFs from inflammatory destruction. Disruption of this signaling axis leads to skin dysbiosis and HF destruction mediated by innate lymphoid cells (53). However, a recent study illustrated that *FGFR2* variants resulted in the activation of EGFR and Notch signaling pathways, along with ADAM10, in an ADAM17-dependent manner (67). Although the molecular mechanisms underlying the crosstalk between ADAM17- and ADAM10-mediated Notch signaling were unknown, it is possible that aberrant activation of ADAM17/EGFR pathways reduces ADAM10 proteolytic activity or alternatively decreases its substrate accessibility (67). Moreover, ADAM17/EGFR signaling promotes the development of the IRS, which is crucial for hair shaft formation (18). However, our findings demonstrated that *Adam17* variant did not impact the expression or phosphorylation of EGFR in Adam17^D647N/D647N^ mice. Based on these findings, we speculate that the downregulation of Notch signaling observed in our study appears to be a secondary effect resulting from dysfunction in the downstream signaling of ADAM17. Future studies are necessary to investigate whether other signaling pathways also contribute to *ADAM17* variant–mediated hair malformations. Understanding the complex interactions between different signaling pathways and their role in HF development may offer insights into potential therapeutic targets for hair loss and other related disorders.

Collectively, this study offers valuable insights into the posttranslational modification of ADAM17, highlighting its pivotal function in HF development and expanding the range of inherited hypotrichosis disorders. Our discoveries reveal new possibilities for therapeutic targets for hair loss.

## Methods

### Sex as a biological variable.

To ensure the generalizability and relevance of our findings across sexes, we included both male and female human participants, as well as male and female mice, in our study. However, in the mouse portion of the study, sex was not treated as a biological variable because of the absence of observed sex-specific differences in any of the measured endpoints between male and female mice.

### Whole-exome sequencing analysis and pathogenic gene identification.

Whole-exome sequencing analysis and pathogenic gene identification were performed using patients’ blood as described previously (68). Comprehensive sequencing data analysis was performed based on autosomal dominant inheritance. Genome-wide linkage analysis was performed with a total of 5,789 SNP markers; their average genetic and physical distances were 436 kb and 0.56 cM, respectively. In the linkage analysis, we removed SNPs with a call rate less than 90% monomorphic SNPs, and non-Mendelian transmitted markers, and retained a total number of 4,532 informative autosomal SNPs. Multipoint parametric linkage analyses were performed using the MERLIN program version 1.1.2. A fully penetrant autosomal dominant model was used with a rare disease frequency of 0.0001. Critical recombination events of the pedigree members were determined through haplotype construction in MERLIN.

### Generation of Adam17 mutation mice.

*Adam17* (p.D647N) mutation mice were generated using CRISPR/Cas9-mediated genome engineering (Cyagen Biosciences). To introduce the desired mutation, a guide RNA targeting the mouse *Adam17* gene, a donor oligo carrying the p.D647N (GAC to AAC) mutation, a synonymous mutation (p. R651= [CGA to AGG]), and Cas9 were coinjected into fertilized mouse eggs, resulting in targeted knockin offspring. F_0_ founder animals were identified by PCR and subsequent sequence analysis. The founder animals were then bred to wild-type mice to assess germ line transmission and F_1_ animal generation. Heterozygous F_1_ mice were intercrossed to generate F_2_ mice. To identify Adam17-mutant mice, genomic tail DNA was isolated and subjected to routine genotyping by PCR and subsequent sequence analysis. The primers utilized for PCR and Sanger sequencing are provided in Supplemental Table 3. All animals were group-housed under 12-hour light/12-hour dark cycle with food and water ad libitum.

### Cell culture.

HaCaT cell lines were purchased from Chinese Academy of Sciences Shanghai Branch Cell Bank. Cells were cultured in DMEM (Gibco), which was supplemented with 10% fetal bovine serum (Gibco) and 1% penicillin-streptomycin (Gibco).

### Tyramide signal amplification multiple immunofluorescence assay.

Tyramide signal amplification multiple immunofluorescences staining experiments were conducted following the manufacturer’s instructions (Shanghai Recordbio Technology). Paraffin-embedded skin sections were hydrated sequentially in xylene and gradient ethanol. Sections underwent antigen retrieval with a citrate repair buffer at pH 6.0 and were blocked with 10% horse serum in PBS at room temperature for 1 hour. Primary antibody was added to the sections and incubated overnight at 4°C. After that, HRP-conjugated secondary antibodies were added and incubated at room temperature for 1 hour. Finally, the sections were incubated with indicated tyramide fluorescence dyes for 15 minutes at room temperature to complete the fluorescence staining of multiple antibodies.We repeated the above steps starting from the antigen retrieval step for the next primary antibody. The antibodies and their dilutions used in this study were listed in Supplemental Table 4.

### Flow cytometry.

Skin epidermal cell suspensions were obtained from defined areas of back skin. Skin was minced and incubated overnight in HBSS buffer (Thermo Fisher Scientific) containing 5 mg/mL dispase II (Yeasen). Next day, the separated epidermal tissue was added to 0.05% trypsin (Thermo Fisher Scientific) with 100 μg/mL DNase I (MilliporeSigma) and agitated for 10 minutes. Thereafter, suspension cells were passed through a 75 μm sieve (MilliporeSigma) and sequentially stained with Fixable Viability Dye eFluor 455UV, anti-CD34 eFluor 660, anti-ITGA6 PE, and anti-Ki67 FITC antibodies. Data were acquired using a BD LSRFortessa X-20 instrument and analyzed on FlowJo software.

### Western blot.

Total protein was extracted from cells and tissues by using RIPA lysis buffer, then subjected to separation using sodium dodecyl sulfate-polyacrylamide gel electrophoresis and subsequently electro-transferred onto a polyvinylidene difluoride membrane (MilliporeSigma), in accordance with previously established protocols (69). Thereafter, the membrane was blocked with 3% BSA at room temperature for 60 minutes, then incubated overnight with primary antibodies as indicated. Following this, the membrane was incubated with secondary antibodies for 1.5 hours. The details of the antibodies and their respective dilutions used in this study are provided in Supplemental Table 4.

### Quantitative RT-PCR.

Total RNA was extracted from mouse skin or cells using the RNeasy kit, following the manufacturer’s instructions (QIAGEN). The mRNA was reverse-transcribed into cDNA using the PrimeScript RT Reagent kit (Takara). Gene expression was quantified in real time using the SYBR Premix Ex Taq kit (Takara) on a QuantStudio 3 (Applied Biosystems) according to the manufacturer’s protocols. Primers used for RT-PCR are provided in Supplemental Table 3. Relative expression was normalized to levels of β-actin.

### In vivo ubiquitination assays.

HEK293T cells (Chinese Academy of Sciences Shanghai Branch Cell Bank) were transfected with His-tagged ubiquitin and indicated plasmids. After 24 hours, MG-132 (MedChemExpress, MCE) was added for 8 hours, followed by cell lysis in IP lysis buffer. The lysates were precleared with protein A/G beads (Santa Cruz Biotechnology) for 3 hours, and the cleared supernatants were incubated with HA-tagged antibodies and protein A/G beads at 4°C for 16 hours. Following this, the pulldown products were washed 3 times with IP lysis buffer and detected by immunoblotting. The details of the antibodies and their respective dilutions used in this study are provided in Supplemental Table 4.

### Protein stability and degradation.

Cycloheximide chase assays were performed on HaCaT cells seeded into 12-well plates to 80%–90% confluence. The following day, cells were treated with 50 μM cycloheximide (MCE) for 2, 4, 6, 8, and 12 hours, respectively. The cells were then lysed with RIPA lysis buffer and analyzed by Western blot.

Protein degradation by proteasome pathway was investigated in HaCaT cells seeded in 12-well plates at 80%–90% confluence. The next day, cells were treated with 10 μM MG132 (MCE) for 2, 4, 6, 8, and 12 hours. The cells were then lysed with RIPA buffer and analyzed using Western blotting.

Protein degradation by autophagy pathway was investigated in HaCaT cells seeded in 12-well plates at 80%–90% confluence. The next day, cells were treated with 50 μM hydroxychloroquine (MCE) for 2, 4, 6, 8, and 12 hours. The cells were then lysed with RIPA buffer and analyzed using Western blotting.

### Pulldown assay.

His-tagged TRIM47 proteins were expressed in bacterial BL21 cells (ABclonal Technology). Following bacterial lysis, His-tagged TRIM47 was purified using Ni-NTA beads (Thermo Fisher Scientific). To investigate the direct interaction between TRIM47 and ADAM17, purified TRIM47 was incubated with in vitro-translated wild-type or mutant ADAM17 overnight at 4°C. After washing with ice-cold buffer, proteins were eluted from the beads and detected by immunoblotting.

### Statistics.

All data were analyzed statistically using GraphPad Prism Version 8.0. All results were presented as mean ± SD. Shapiro-Wilk test was performed to check the data distribution. Unpaired 2-tailed *t* tests or Mann-Whitney 2-tailed tests were utilized for comparisons between 2 groups. For comparison among multiple groups, ordinary 1-way ANOVA with Dunnett’s post hoc or Kruskal-Wallis test followed by Dunn’s post hoc was used based on the Gaussian or non-Gaussian distribution of the data. *P* < 0.05 (2 sided) was considered statistically significant.

### Study approval.

This study was approved by Xinhua Hospital, Shanghai Jiaotong University School of Medicine, and was conducted in accordance with the Declaration of Helsinki and the Department of Health and Human Services Belmont Report. All participants in this study were thoroughly informed about the study’s objectives, methods, and potential risks and the benefits they could derive from it. Consequently, all patients voluntarily signed a written informed consent form, agreeing to participate in the study and authorizing the use of their tissue samples and photographs for clinical research and potential academic publication. All animal experiments adhered to the regulations specified in the *Guide for the Care and Use of Laboratory Animals* (National Academies Press, 2011). Approval for conducting animal studies was obtained from the Animal Studies Committee at Xinhua Hospital Affiliated to Shanghai Jiaotong University School of Medicine.

### Data availability.

The data sets supporting the conclusions of this article are included within the article and its additional files. All supporting data values pertinent to the main manuscript and supplemental materials, including numerical values for all data points depicted in graphs and the underlying values for any reported means, were comprehensively included in the Supporting Data Values file. In this study, the produced data sets are available in the following databases: The mass spectrometry proteomics data and protein interaction AP-MS data have been submitted to the ProteomeXchange Consortium (https://proteomecentral.proteomexchange.org) via the iProX partner repository, with the data set identifiers PXD049458 and PXD049459, respectively. Human variation data have been submitted to CNGB Sequence Archive (https://db.cngb.org/cnsa/) with the data set identifier sub052490.

## Author contributions

XW and ML designed and conceived this project and developed methodology. XW, CP, QZ, WH, KZ, YW, AZ, and QC performed experiments and generated data. JW and LZ conducted collection of clinical patient data and relevant specimens. XW, PS, and RC analyzed and interpreted data. XW and CP wrote the draft. ML, SZ, ZY, and HZ revised and finalized the manuscript. All authors contributed to and approved the manuscript. XW, CP, LZ, JW, and QZ contributed equally to this work. The order of co–first authors is determined based on the contributions of each author.

## Supplementary Material

Supplemental data

Unedited blot and gel images

Supporting data values

## Version 1 05/21/2024

In-Press Preview

## Version 2 07/08/2024

Electronic publication
