# Supplementary material for: ADAM17 variant causes hair loss via ubiquitin ligase TRIM47–mediated degradation
Source: JCI Insight. 2024 May 21;9(13):e177588. doi: 10.1172/jci.insight.177588 (PMC11383180; doi:10.1172/jci.insight.177588)
Supplement: Supplemental data [file jciinsight-9-177588-s036.pdf]

Supplementary Materials for

ADAM17 variant causes hair loss via ubiquitin ligase TRIM47 mediated degradation.

[*]**Correspondence:** mingli@fudan.edu.cn; zhanghui@xinhuamed.com.cn; zhangsi@fudan.edu.cn

Table of contents

Supporting Fig. 1...2-3

Supporting Fig. 2...4

Supporting Fig. 3...4-5

Supporting Fig. 4...6-7

Supporting Fig. 5...7-8

Supporting Fig. 6...8-9

Supporting Fig. 7...9-10

Supporting Fig. 8...10-11

Supporting Table 1...12-15

Supporting Table 2...15-18

Supporting Table 3...18-20

Supporting Table 4...21-23

Reference……………………………………………………………………….24

Supporting Figures and Figure Legends

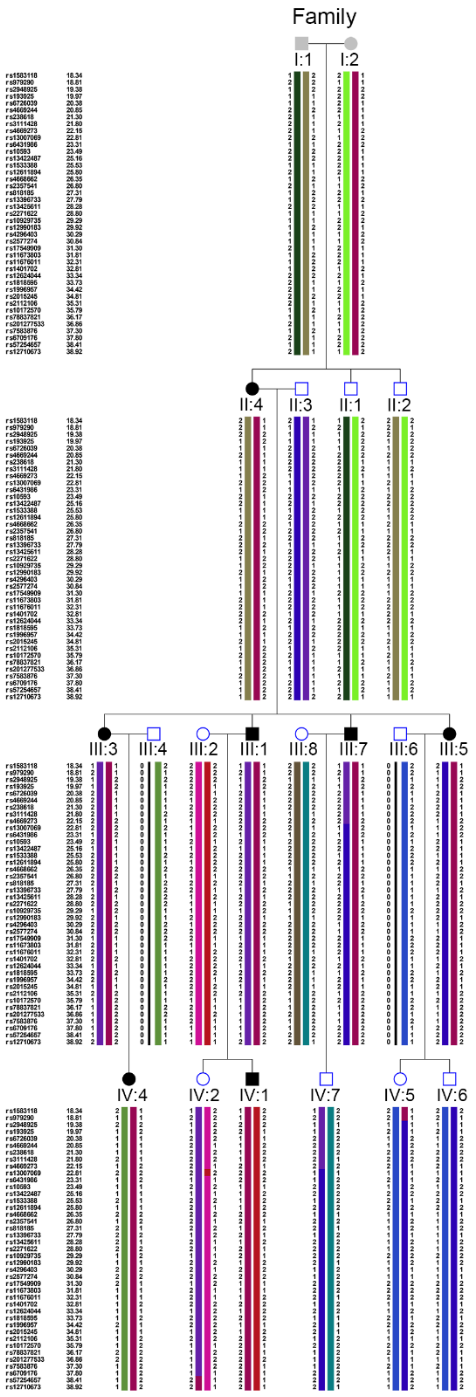

Supporting Fig. 1 *ADAM17* variants leads to autosomal dominant hypotrichosis with woolly hair.

Haplotype analysis narrowed the candidate region to 19.6cM between markers

rs979290 and rs57254657, which contained 126 annotated genes.

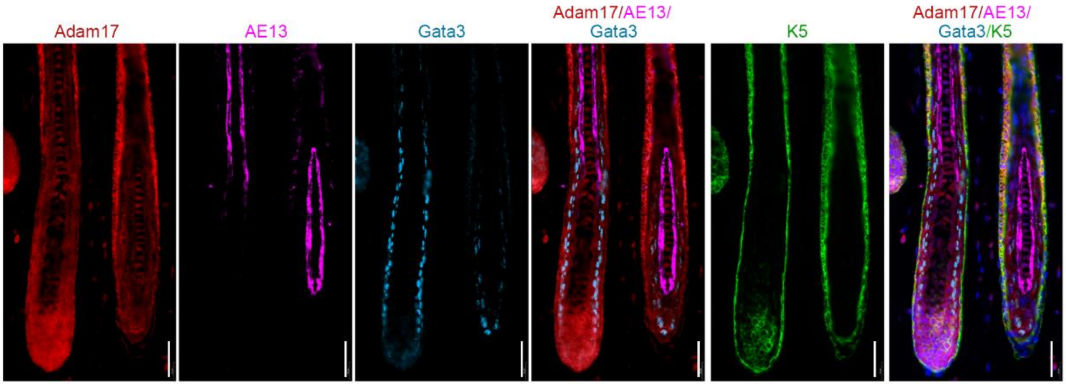

Supporting Fig. 2 Adam17 exhibits high expression in the hair follicles of mice.

Immunofluorescence demonstrated that Adam17 was predominantly expressed in the hair cortex, inner root sheath (IRS), and outer root sheath (ORS) of mice hair follicle. The Gata3 antibody was utilized to label the IRS, while the AE13 antibody was employed to label the keratins in the hair cortex. Additionally, the K5 antibody was used to label the ORS. Scale bars, 40 μm.

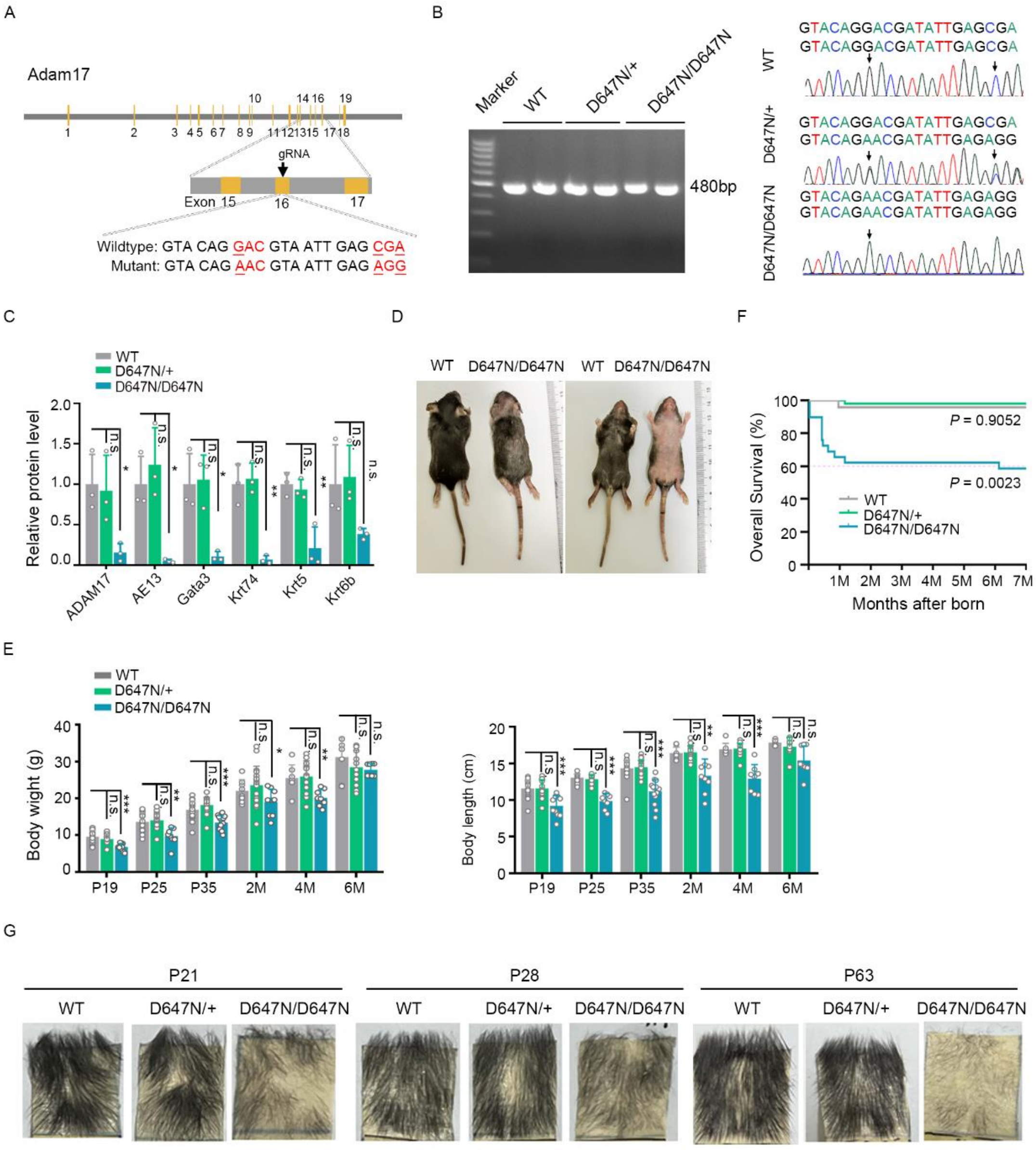

Supporting Fig. 3 ***Adam17*** **(p.D647N) variant leads to developmental delay and severe skin inflammation in mice.**

(**A**) Schematic diagram of the strategy to generate mouse model with ADAM17 point mutation by CRISPR/Cas9 mediated genome engineering. The p.D647N (GAC to AAC) in donor oligo was introduced into exon 16 by homology-directed repair. One synonymous mutations p.R651 (CGA to AGG) would also be introduced to prevent the binding and re-cutting of the sequence by gRNA after homology-directed repair. (**B**)

Genotype were identified by PCR followed by sequence analysis. Both wild-type and mutant mice had 480 bp band. Subsequently, the mouse genotype was confirmed using Sanger sequencing. (WT) wild-type littermates; (D647N/+) heterozygous mice; (D647N/D647N) homozygous mice. (**C**) Quantification of the immunoblotting results corresponding to Figure 2H showing a substantial decrease in IRS markers within the hair follicles of Adam17$^{D647N/D647N}$ mice. (n = 3 biological replicates). (**D**) Adam17$^{D647N/D647N}$ mice showed severe skin inflammation. (**E**) *Adam17* (p.D647N) variants led to developmental delay in mice. Left panel: quantification of mice weight. Right panel: quantification of mice length. (n = 8-29 biological replicates). (**F**) *Adam17* (p.D647N) variants was associated with significantly worse overall survival of mice. Adam17$^{D647N/D647N}$ mice might die within the first month after birth due to either hydrocephalus or severe skin inflammation. (n = 23-51 biological replicates). (**G**) Tape was affixed to the hair coat and peeled off during different hair cycles. All experiments were repeated three times. Results were expressed as mean ± SD; n.s., not significant; **P* < 0.05; ***P* < 0.01; ****P* < 0.001; One-way ANOVA test (C, E); Kruskal-Wallis test (E); Brown-Forsythe and Welch ANOVA tests (E); log-rank t test (F).

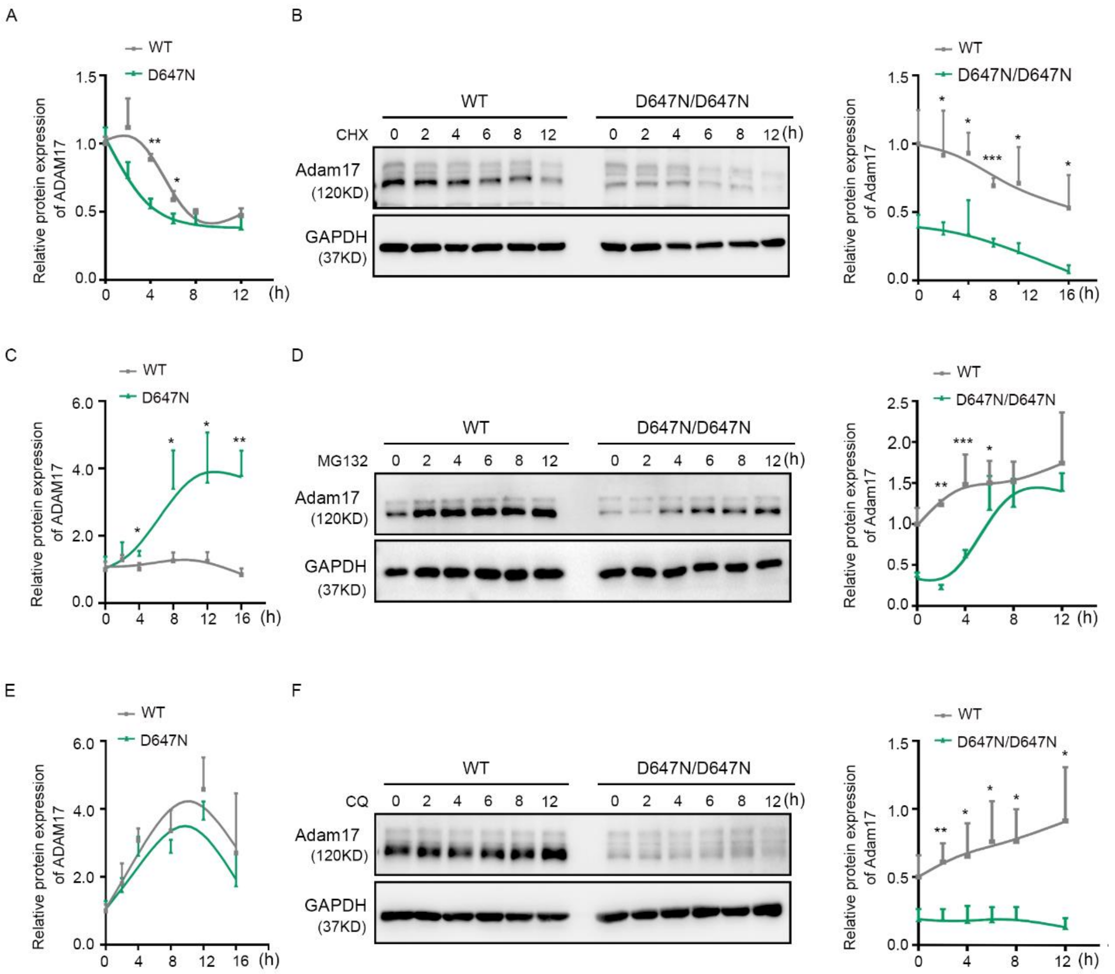

Supporting Fig. 4 *Adam17* (p.D647N) variant decreases its protein stability owing to enhanced auto-ubiquitination.

(**A**) Quantification of the immunoblotting results corresponding to Figure 4G. (n = 3 biological replicates). (**B**) Cycloheximide (CHX) chase analysis showed that *Adam17* (p.D674N) mutation induced rapid degradation of Adam17 in primary mouse fibroblasts cells. (n = 3 biological replicates) (**C**) Quantification of the immunoblotting results corresponding to Figure 4H. (n = 3 biological replicates). (**D**) *Adam17* (p.D647N) mutation resulted in heightened degradation of Adam17 though proteasome pathway in in primary mouse fibroblasts cells. (n = 3 biological replicates) (**E**) Quantification of

the immunoblotting results corresponding to Figure 4I. (n = 3 biological replicates). (**F**) *Adam17* (p.D647N) mutation had no bearing on the degradation of Adam17 through the autophagy pathway in primary mouse fibroblasts cells. (n = 3 biological replicates). All experiments were repeated three times. Results were expressed as mean ± SD, n.s., not significant; **P* < 0.05; ***P* < 0.01; ****P* < 0.001, Unpaired two-tailed t test (A, B, C, D, E, F); Mann-Whitney test (B, D, E, F).

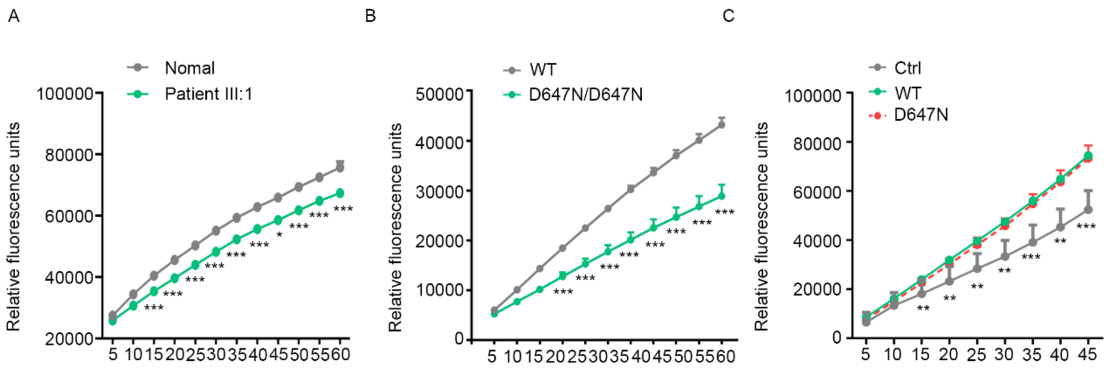

Supporting Fig. 5 *ADAM17* (p.D647N) variant does not impact its shedding activity.

(**A**) The shedding activity of ADAM17 in patients' scalp tissue was significantly lower than that in normal controls. (n = 4 biological replicates) (**B**) The shedding activity of Adam17 in primary cultured mouse skin fibroblast cells form Adam17$^{D647N/D674N}$ mice was significantly reduced compared to the wild-type mice (n = 5-6 biological replicates). (**C**) No significant difference in the shedding activity of ADAM17 between the wildtype and ADAM17 (p.D647N) mutant HaCaT cells (n = 6 biological replicates). All experiments were repeated three times. Results were expressed as mean ± SD, n.s., not significant; ***P* < 0.01; ****P* < 0.001, Unpaired two-tailed t test (A, B); Mann-

Whitney test (A); Unpaired t test with Welch's correction (B); One-way ANOVA(C); Kruskal-Wallis test (C).

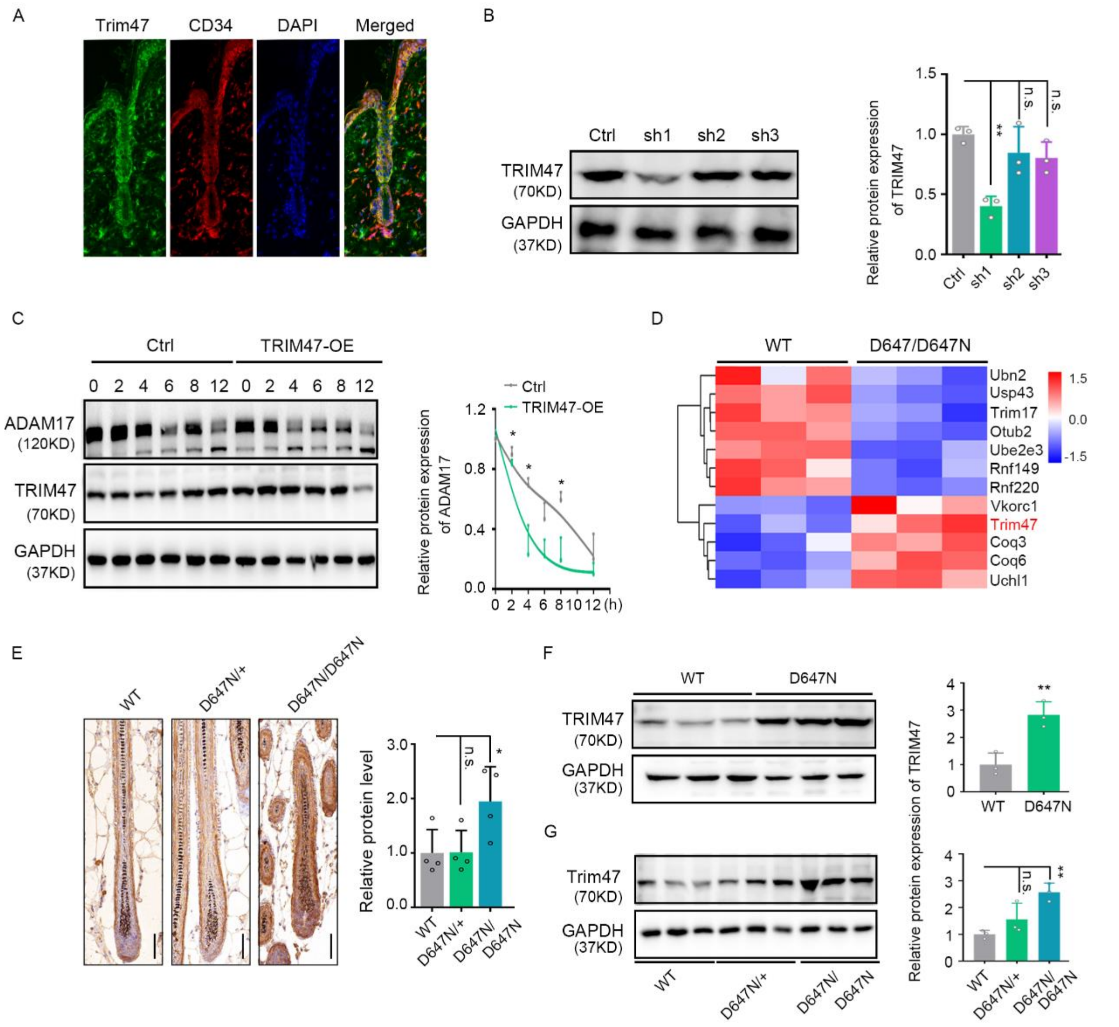

Supporting Fig. 6 TRIM47 is identified as an ADAM17 specific E3 ubiquitin ligase. (**A**) TRIM47 showed high expression in hair follicle stem cells. Scale bars, 40 µm. (**B**) The efficiency of *TRIM47*-knockdown in HaCaT cells was detected by immunoblotting. (n = 3 biological replicates). (**C**) Overexpression of TRIM47 promoted the proteasomal degradation of ADAM17. Left panel：Representative immunoblot images of the ADAM17 protein levels during CHX chase assays. Right panel: quantification of the

immunoblotting results corresponding to the left panel (n = 3 biological replicates). (**D**) Heatmap of proteins associated with ubiquinone and other terpenoid-quinone biosynthesis pathways from Proteomics-seq. (**E**) Immunohistochemical staining showed that *Adam17* (p.D647N) mutation led to up-regulation the expression of TRIM47 in mice. Left panel: representative images of immunohistochemical staining. Right panel: statistical results of the left panel. (n = 4 biological replicates). (**F**) TRIM47 protein level was significantly increased in *ADAM17* (p.D647N) mutant HaCaT cells. Left panel: representative images of immunoblotting. Right panel: statistical results of the left panel. (n = 3 biological replicates). (**G**) *Adam17* (p.D647N) mutation resulted in an up-regulation of Trim47 expression in the skin of mice. Left panel: representative images of immunoblotting. Right panel: statistical results of the left panel. (n = 3 biological replicates). All experiments were repeated three times. Results were expressed as mean ± SD, n.s., not significant; **P* < 0.05; ***P* < 0.01, Unpaired two-tailed t test (C, F); One-way ANOVA test (B, E, G).

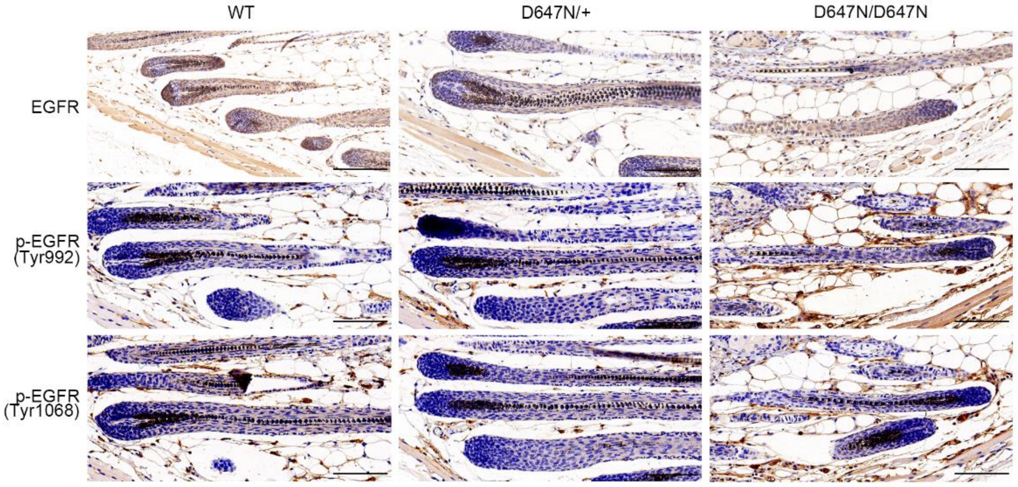

Supporting Fig. 7 *Adam17* (p.D647N) variant does not impede EGFR signaling

pathway in mice.

Immunohistochemical staining revealed that *Adam17* (p.D647N) mutation does not influence the expression of epidermal growth factor receptor (EGFR), as well as phosphorylated EGFR at Tyrosine 992 (p-EGFR (Try992)) and Tyrosine 1068 (p-EGFR (Try1068)) in Adam17$^{D647N/D647N}$ mice. Scale bar, 100 μm.

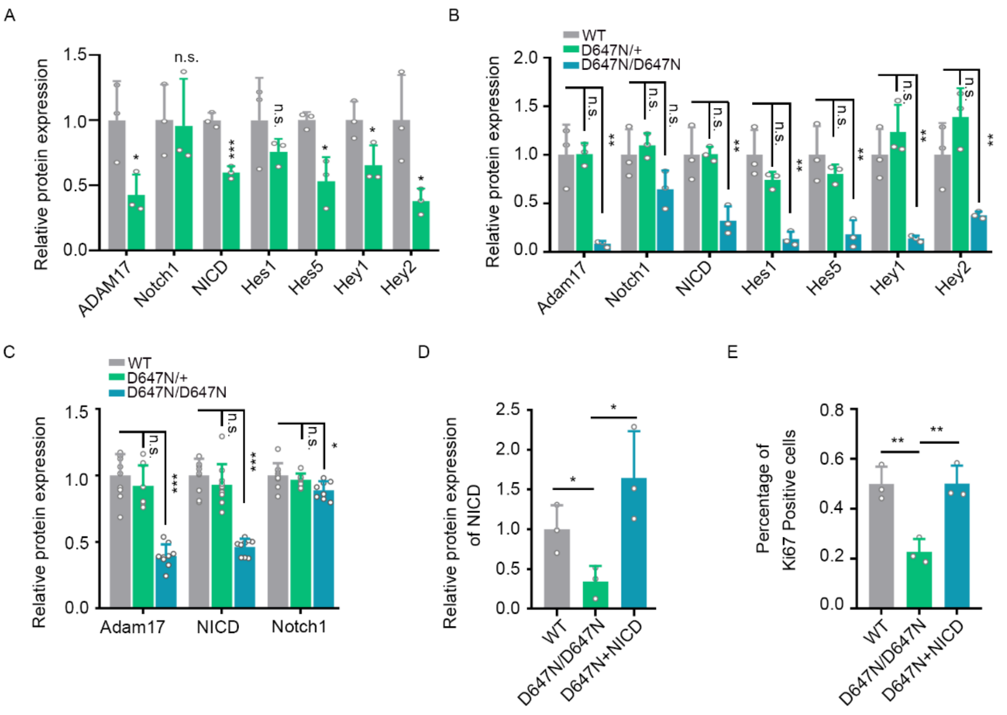

Supporting Fig. 8 *ADAM17* (p.D647N) variant affects Notch signaling pathway.

(**A**) Quantification of the immunoblotting results corresponding to Figure 7A showing the effect of *ADAM17* (p.D647N) mutation on protein levels of key molecules involved in Notch signaling in the skin biopsy of the patient. (n = 3 biological replicates). (**B**) Quantification of the immunoblotting results corresponding to Figure 7B showing the effect of *ADAM17* (p.D647N) mutation on protein levels of key molecules involved in Notch signaling in the skin tissues of mice. (n = 3 biological replicates). (**C**)

Quantification of the immunohistochemical staining images corresponding to Figure 7D. (n = 8-10 biological replicates). (**D**) The immunoblotting results corresponding to Figure 7G were quantified to determine the efficiency of NICD overexpression in primary fibroblasts. (n = 3 biological replicates). (**E**) The percentage of Ki67-positive cells was quantified for Figure 7H. (n = 3 biological replicates). All experiments were repeated three times. Results were expressed as mean ± SD, n.s., not significant; *$P < 0.05$; **$P < 0.01$; ***$P < 0.001$; Unpaired two-tailed t test (A, D); One-way ANOVA test (B, C, E); Kruskal-Wallis test (C).

Supporting Tables

Supporting Table 1. Summary of mass spectrometry (MS) analysis of ADAM17 interactors.

Identified proteins	PSM WT	PSM (D647N)	PSM (D647N) / (WT)	Protein Accession	Protein Description
**TRIM47**	**28**	**139**	**4.964285714**	**Q96LD4**	**E3 ubiquitin-protein ligase TRIM47**
ALBU	105	113	1.076190476	P02768	Albumin
PTPRZ	100	58	0.58	P23471	Receptor-type tyrosine-protein phosphatase zeta
M4K2	28	56	2	Q12851	Mitogen-activated protein kinase kinase kinase kinase 2
NDST1	27	55	2.037037037	P52848	Bifunctional heparan sulfate N-deacetylase/N-sulfotransferase 1
DIP2A	27	55	2.037037037	Q14689	Disco-interacting protein 2 homolog A
AT10B	27	55	2.037037037	O94823	Phospholipid-transporting ATPase VB
**ADAM17**	**40**	**41**	**1.025**	**P78536**	**Disintegrin and metalloproteinase domain-containing protein 17**
GRP75	70	38	0.542857143	P38646	Stress-70 protein, mitochondrial
K2C1	34	36	1.058823529	P04264	Keratin, type II cytoskeletal 1
KCC2D	8	36	4.5	Q13557	Calcium/calmodulin-dependent protein kinase type II

					subunit delta
HS71A	49	32	0.653061224	P0DMV8	Heat shock 70 kDa protein 1A
HS71B	49	32	0.653061224	P0DMV9	Heat shock 70 kDa protein 1B
KCC2G	6	30	5	Q13555	Calcium/calmodulin-dependent protein kinase type II subunit gamma
K1C10	33	29	0.878787879	P13645	Keratin, type I cytoskeletal 10
HSP7C8	43	25	0.581395349	P11142	Heat shock cognate 71 kDa protein
K22E	24	24	1	P35908	Keratin, type II cytoskeletal 2 epidermal
GDE	20	22	1.1	P35573	Glycogen debranching enzyme
K1C9	26	17	0.653846154	P35527	Keratin, type I cytoskeletal 9
KPBB	16	17	1.0625	Q93100	Phosphorylase b kinase regulatory subunit beta
GTF2I	16	16	1	P78347	General transcription factor II-I
BIP	26	15	0.576923077	P11021	Endoplasmic reticulum chaperone BiP
BTBD8	25	15	0.6	Q5XKL5	BTB/POZ domain-containing protein 8
NONO	13	15	1.153846154	Q15233	Non-POU domain-containing octamer-binding protein
HSP72	20	14	0.7	P54652	Heat shock-related 70 kDa protein 2
SLK	12	12	1	Q9H2G2	STE20-like serine/threonine-protein kinase
UTRN	12	12	1	P46939	Utrophin
CYTSB	12	12	1	Q5M775	Cytospin-B
DMD	12	12	1	P11532	Dystrophin

BACD3	12	12	1	Q9H3F6	BTB/POZ domain-containing adapter for CUL3-mediated RhoA degradation protein 3
SFPQ	20	11	0.55	P23246	Splicing factor, proline- and glutamine-rich
DHX29	1	11	11	Q7Z478	ATP-dependent RNA helicase DHX29
IBTK	18	10	0.555555556	Q9P2D0	Inhibitor of Bruton tyrosine kinase
GSTP1	17	10	0.588235294	P09211	Glutathione S-transferase P
K2C4	14	10	0.714285714	P19013	Keratin, type II cytoskeletal 4
G3P	9	10	1.111111111	P04406	Glyceraldehyde-3-phosphate dehydrogenase
HS90B	11	10	0.909090909	P08238	Heat shock protein HSP 90-beta
TCP4	7	10	1.428571429	P53999	Activated RNA polymerase II transcriptional coactivator p15
RL4	7	10	1.428571429	P36578	60S ribosomal protein L4
AT8A1	4	10	2.5	Q9Y2Q0	Phospholipid-transporting ATPase
CLNK	3	10	3.333333333	Q7Z7G1	Cytokine-dependent hematopoietic cell linker
T132D	3	10	3.333333333	Q14C87	Transmembrane protein 132D
LCP2	3	10	3.333333333	Q13094	Lymphocyte cytosolic protein 2
DYST	3	10	3.333333333	Q03001	Dystonin
CFA69	3	10	3.333333333	A5D8W1	Cilia- and flagella-associated protein 69
KPB2	17	9	0.529411765	P46019	Phosphorylase b kinase regulatory subunit alpha, liver isoform

WIPI3	12	9	0.75	Q5MNZ6	WD repeat domain phosphoinositide-interacting protein 3
CPVL	12	9	0.75	Q9H3G5	Probable serine carboxypeptidase CPVL
NUCL	11	9	0.818181818	P19338	Nucleolin
RO52	10	9	0.9	P19474	E3 ubiquitin-protein ligase TRIM21
RL6	7	9	1.285714286	Q02878|	60S ribosomal protein L6
ADT2	7	9	1.285714286	P05141	ADP/ATP translocase 2
PIMT	9	8	0.888888889	P22061	Protein-L-isoaspartate(D-aspartate) O-methyltransferase
K1C14	8	8	1	P02533	Keratin, type I cytoskeletal 14
DCTN5	15	4	0.266666667	Q9BTE1	Dynactin subunit 5
SEPT7	14	4	0.285714286	Q16181	Septin-7
GRN	11	4	0.363636364	P28799	Progranulin
PRDX1	9	4	0.444444444	Q06830	Peroxiredoxin-1
ATPA	7	4	0.571428571	P25705	ATP synthase subunit alpha, mitochondrial
NPM	7	4	0.571428571	P06748	Nucleophosmin
PHB1	7	4	0.571428571	P35232	Prohibitin 1
DCTN5	15	4	0.266666667	Q9BTE1	Dynactin subunit 5

Abbreviations: PSM, peptide spectrum match.

Supporting Table 2. Classification of non-syndromic congenital hypotrichosis.

Name	OMIM	Inheritance	Gene	Locus	Phenotype	Reference
HYPT1 (HHS1)	605389	AD	*APCDD1*	18p11.22	Sparse, short and thin hair on scalp and body. Eyebrows, eyelashes and beard hair are normal	(1)
HYPT2 (HHS2)	146520	AD	*CDSN*	6p21.33	Normal hair at birth, sparse hair on scalp and body, scalp hair growth retardation with diffuse hair loss. Eyebrows, eyelashes and beard hair are normal	(2)
HYPT3 (ADWH1)	613981	AD	*KRT74*	12q13.13	Sparse scalp hair with wooly hair. Eyebrows, eyelashes, and beard hair are normal	(3)
HYPT4 (MUHH1)	146550	AD	*U2HR*	8p21.3	Sparse and fragile hair with hair growth retardation	(4)
HYPT5 (MUHH2)	612841	AD	*EPS8L3*	1p13.3	Sparse to absent scalp hair at birth. Irregular hair in childhood. Thin eyebrows and eyelashes	(5)
HYPT6 (LAH1)	697903	AR	*DSG4*	18q12.1	Localized sparse scalp hairs, absence of eyebrows and eyelashes. axillary and pubic hairs, follicular papules on scalp are normal	(6)
HYPT7 (ARWH2, LAH2)	604379	AR	*LIPH*	3q27.2	Localized sparse, thin, fragile and short scalp hair with wooly, slightly colored hair. Normal sparse eyebrows, eyelashes, axillary and body hair	(7)

HYPT8 (ARWH1, LAH3)	278150	AR	*LPAR6*	13q14.2	Slow growing hair with woolly hair, normal to sparse eyebrows and eyelashes, popular lesions on the occipital region	(8)
HYPT9 (LAH4)	614237	AR	unknown	10q11.23-q22.3	Localized sparse and slightly brown hair on scalp, arms and legs. Normal eyebrows and eyelashes	(9)
HYPT10 (LAH5)	614238	AR	unknown	7q22.3-21.3	Absent hair at birth. Sparse scalp, eyebrows, eyelashes and body hairs with papules on scalp	(10)
HYPT11 (HHS3)	615059	AD	*SNRPE*	1q32.1	Sparse to absent scalp, body hair and eyebrows, normal pubic hair	(11)
HYPT12 (HHS4)	615885	AD	*RPL21*	13q12.2	Hair loss began at 2–6 months, absent to sparse scalp hair, eyebrows, eyelashes, body hair, axillary and pubic hairs	(12)
HYPT13 (ADWH2)	615896	AD	*KRT71*	12q13.13	Short sparse hair with woolly hair. Sparse eyebrows and eyelashes	(13)
HYPT14 (HHS5)	618275	AR	*LSS*	21q22.3	Scalp hair loss and partial loss of the eyebrows and eyelashes with paucity of body hair	(14)
ARWH3	616760	AR	*KRT25*	17q21.2	Soft, short and sparse hairs on the scalp. Normal eyebrows, but sparse eyelashes	(15)
–	–	AD	*KRT25*	17q21.2	Sparse, soft, and curled scalp hairs	(16)
–	–	AR	*C3ORF52*	3q13.2	Sparse scalp, eyebrows, and eyelashes	(17)

**ADWH3**	**–**	**AD**	***ADAM17***	**2p25.1**	**Sparse scalp, eyebrows and eyelashes with woolly hair**	**Our study**

Abbreviations: HYPT, hypotrichosis; AD, autosomal dominant; AR, autosomal recessive; HHS, hereditary hypotrichosis simplex; MUHH, Marie Unna hereditary hypotrichosis; LAH, localized autosomal recessive hypotrichosis; ARWH, autosomal recessive woolly hair/hypotrichosis; OMIM, Online Mendelian Inheritance in Man.

Supporting Table 3. Primers used for PCR.

Primer name	Primer sequence 5'-3'
Genotyping Primer F	GGATGTATTGTGACAGTGCTAGTG
Genotyping Primer R	GAAACAAATGCTGGAGTCCCTGAA
Sequencing Primer	GGATGTATTGTGACAGTGCTAGTG
Trim47 sh1 Primer F	CCGGGCCGTAGACAACACGTGTGTACTCGAGTACCACACGTGTTGTCTACGGCTTTTTG
Trim47 sh1 Primer R	AATTCAAAAATAGACAACACGTGTGTACTCGAGTACACACGTGTTGTCTACGGC
Trim47 sh2 Primer F	CCGGGCCAGCAAGTGTGACAGTCATCTCGAGATGACTGTCACACTTGCTGGCTTTTTG
Trim47 sh2 Primer R	AATTCAAAAAGCCAGCAAGTGTGACAGTCATCTCGAGATGACTGTCACACTTGCTGGC
Trim47 sh3 Primer F	CCGGGCCACCTTTACTCTGCTCTATCTTCGAGATAGAGCAGAGTAAAGGTGGCTTTTTG
Trim47 sh3 Primer R	AATTCAAAAAGCCACCTTTACTCTGCTCTATCTTCGAGATAGAGCAGAGTAAAGGTGGC
ADAM17 human F	GTGGATGGTAAAAACGAAAGCG

ADAM17 human R	GGCTAGAACCCTAGAGTCAGG
β-actin human F	CATGTACGTTGCTATCCAGGC
β-actin human R	CTCCTTAATGTCACGCACGAT
ADAM17 mouse F	ACCACTTTGGTGCCTTTCGT
ADAM17 mouse R	GTCGCAGACTGTAGATCCCTT
Notch1 mouse F	GATGGCCTCAATGGGTACAAG
Notch1 mouse R	TCGTTGTTGTTGATGTCACAGT
Notch2 mouse F	GAGAAAAACCGCTGTCAGAATGG
Notch2 mouse R	GGTGGAGTATTGGCAGTCCTC
Notch3 mouse F	AGTGCCGATCTGGTACAACTT
Notch3 mouse R	CACTACGGGGTTCTCACACA
Notch4 mouse F	CTCTTGCCACTCAATTTCCCT
Notch4 mouse R	TTGCAGAGTTGGGTATCCCTG
Hey1 mouse F	CCGACGAGACCGAATCAATAAC
Hey1 mouse R	TCAGGTGATCCACAGTCATCTG
Hey2 mouse F	CGCCCTTGTGAGGAAACGA
Hey2 mouse R	CCCAGGGTAATTGTTCTCGCT
Hes1 mouse F	TCAACACGACACCGGACAAAC
Hes1 mouse R	ATGCCGGGAGCTATCTTTCTT
Hes5 mouse F	AGTCCCAAGGAGAAAAACCGA

Hes5 mouse R	GCTGTGTTTCAGGTAGCTGAC
Ptcra mouse F	CGTCAGGTGTCAGGCTCTAC
Ptcra mouse R	GTGAAGGCGTCTAGGGCAC
β-actin mouse F	GGCTGTATTCCCCTCCATCG
β-actin mouse R	CCAGTTGGTAACAATGCCATGT

Supporting Table 4. Antibodies and dilutions used in this study.

Antibody	Dilutions for					Company	Catalogue No.
	WB	IF	IP	IHC	FACS		
ADAM17	1:1000	1:100	/	1:100	/	Abcam	AB39163
ADAM17	1:1000	/	/	/	/	Abclonal	A0821
TRIM47	1:2000	1:100	/	1:100	/	Abclonal	A17803
His-Tag	1:2000	/	/	/	/	Cell Signaling Technology	12698P
Gata3	1:2000	1:500	/	/	/	Cell Signaling Technology	5852T
AE13	1:2000	1:200	/	/	/	Abcam	ab16113
K5	1:2000	1:500	/	/	/	Abcam	Ab52635

Krt74	1:1000	/	/	/	/	Thermo fisher scientific	PA5-45864
Krt6a	1:1000	/	/	/	/	Proteintech	17391-1-AP
CD200	/	1:50	/	/	/	Abclonal	A21226
HA-Tag (C29F4)	1:2000	1:200	1:100	/	/	Cell Signaling Technology	3724S
Notch1	1:1000	/	/	1:200	/	Cell Signaling Technology	3608S
Cleaved Notch1	1:1000	1:200	/	1:200	/	Cell Signaling Technology	4147S
Hey1	1:1000	/	/	/	/	Abclonal	A16110
Hey2	1:1000	/	/	/	/	Abclonal	A15143
Hes1	1:1000	/	/	/	/	Abclonal	A16110
Hes5	1:1000	/	/	/	/	Abcam	ab194111
Ki67	/	1:300	/	/	/	Cell Signaling Technology	9449T
Ubiquitin	1:2000					Santa cruz	sc-8017
Fixable Viability Dye eFluor 455UV	/	/	/	/	1:1000	Ebioscience	65-0868-14
CD34 Monoclonal Antibody eFluor 660	/	/	/	/	1:150	ebioscience	50-0341-82
Ki-67 Monoclonal Antibody FITC	/	/	/	/	1:100	ebioscience	11-5698-80

CD49f (Integrin alpha 6) Monoclonal Antibody, PE	/	/	/	/	1:100	ebioscience	12-0495-83
LaminB	1:1000	/	/	/	/	Cell Signaling Technology	17416T
GAPDH	1:2000	/	/	/	/	Yeasen	30203ES50
α-tubulin	1:2000	/	/	/	/	Yeason	
CD34	/	1:100	/	/	/	Abcam	30303ES50
K15	/	1:200	/	/	/	Proteintech	10137-1-AP
HRP-linked Goat Anti Rabbit IgG	1:5000	/	/	/	/	Jackson ImmunoResearch	115-035-003
HRP-linked Goat Anti Mouse IgG	1:5000	/	/	/	/	Jackson ImmunoResearch	111-545-003
Alexa Fluor® 488 Goat Anti Rabbit IgG	/	1:200	/	/	/	Jackson ImmunoResearch	115-605-003
Alexa Fluor® 647 Goat Anti Mouse IgG	/	1:200	/	/	/	Jackson ImmunoResearch	A12380
EGFR	/	/	/	1:200	/	Proteintech	66455-1-Ig
p-EGFR(Try992)	/	/	/	1: 200	/	Cell Signaling Technology	2235T
p-EGFR(Try1068)	/	/	/	1:400	/	Cell Signaling Technology	3777T

Abbreviations: WB, western-blot; IF, Immunofluorescence; IP, Immunoprecipitation; IHC, Immunohistochemistry; FACS, Flow Cytometry.

References:

1. Shimomura Y, Agalliu D, Vonica A, Luria V, Wajid M, Baumer A, et al. APCDD1 is a novel Wnt inhibitor mutated in hereditary hypotrichosis simplex. *Nature.* 2010;464(7291):1043-7.
2. Levy-Nissenbaum E, Betz RC, Frydman M, Simon M, Lahat H, Bakhan T, et al. Hypotrichosis simplex of the scalp is associated with nonsense mutations in CDSN encoding corneodesmosin. *Nature genetics.* 2003;34(2):151-3.
3. Shimomura Y, Wajid M, Petukhova L, Kurban M, and Christiano AM. Autosomal-dominant woolly hair resulting from disruption of keratin 74 (KRT74), a potential determinant of human hair texture. *American journal of human genetics.* 2010;86(4):632-8.
4. Wen Y, Liu Y, Xu Y, Zhao Y, Hua R, Wang K, et al. Loss-of-function mutations of an inhibitory upstream ORF in the human hairless transcript cause Marie Unna hereditary hypotrichosis. *Nature genetics.* 2009;41(2):228-33.
5. Zhang X, Guo BR, Cai LQ, Jiang T, Sun LD, Cui Y, et al. Exome sequencing identified a missense mutation of EPS8L3 in Marie Unna hereditary hypotrichosis. *Journal of medical genetics.* 2012;49(12):727-30.
6. Kljuic A, Bazzi H, Sundberg JP, Martinez-Mir A, O'Shaughnessy R, Mahoney MG, et al. Desmoglein 4 in hair follicle differentiation and epidermal adhesion: evidence from inherited hypotrichosis and acquired pemphigus vulgaris. *Cell.* 2003;113(2):249-60.
7. Shimomura Y, Wajid M, Petukhova L, Shapiro L, and Christiano AM. Mutations in the lipase H gene underlie autosomal recessive woolly hair/hypotrichosis. *The Journal of investigative dermatology.* 2009;129(3):622-8.
8. Nahum S, Morice-Picard F, Taieb A, and Sprecher E. A novel mutation in LPAR6 causes autosomal recessive hypotrichosis of the scalp. *Clinical and experimental dermatology.* 2011;36(2):188-94.
9. Naz G, Ali G, Naqvi SK, Azeem Z, and Ahmad W. Mapping of a novel autosomal recessive hypotrichosis locus on chromosome 10q11.23–22.3. *Human genetics.* 2010;127(4):395-401.
10. Basit S, Ali G, Wasif N, Ansar M, and Ahmad W. Genetic mapping of a novel hypotrichosis locus to chromosome 7p21.3-p22.3 in a Pakistani family and screening of the candidate genes. *Human genetics.* 2010;128(2):213-20.
11. Pasternack SM, Refke M, Paknia E, Hennies HC, Franz T, Schäfer N, et al. Mutations in SNRPE, which encodes a core protein of the spliceosome, cause autosomal-dominant hypotrichosis simplex. *American journal of human genetics.* 2013;92(1):81-7.
12. Zhou C, Zang D, Jin Y, Wu H, Liu Z, Du J, et al. Mutation in ribosomal protein L21 underlies hereditary hypotrichosis simplex. *Human mutation.* 2011;32(7):710-4.
13. Fujimoto A, Farooq M, Fujikawa H, Inoue A, Ohyama M, Ehama R, et al. A missense mutation within the helix initiation motif of the keratin K71 gene

underlies autosomal dominant woolly hair/hypotrichosis. *The Journal of investigative dermatology.* 2012;132(10):2342-9.

14. Romano MT, Tafazzoli A, Mattern M, Sivalingam S, Wolf S, Rupp A, et al. Bi-allelic Mutations in LSS, Encoding Lanosterol Synthase, Cause Autosomal-Recessive Hypotrichosis Simplex. *American journal of human genetics.* 2018;103(5):777-85.
15. Akiyama M. Isolated autosomal recessive woolly hair/hypotrichosis: genetics, pathogenesis and therapies. *Journal of the European Academy of Dermatology and Venereology : JEADV.* 2021;35(9):1788-96.
16. Yu X, Chen F, Ni C, Zhang G, Zheng L, Zhang J, et al. A Missense Mutation within the Helix Termination Motif of KRT25 Causes Autosomal Dominant Woolly Hair/Hypotrichosis. *The Journal of investigative dermatology.* 2018;138(1):230-3.
17. Malki L, Sarig O, Cesarato N, Mohamad J, Canter T, Assaf S, et al. Loss-of-function variants in C3ORF52 result in localized autosomal recessive hypotrichosis. *Genetics in medicine : official journal of the American College of Medical Genetics.* 2020;22(7):1227-34.
