# Supplementary material for: ADAM17 variant causes hair loss via ubiquitin ligase TRIM47–mediated degradation
Source: JCI Insight. 2024 May 21;9(13):e177588. doi: 10.1172/jci.insight.177588 (PMC11383180; doi:10.1172/jci.insight.177588)
Supplement: Unedited blot and gel images [file jciinsight-9-177588-s037.pdf]

Full unedited gel for Figure2G

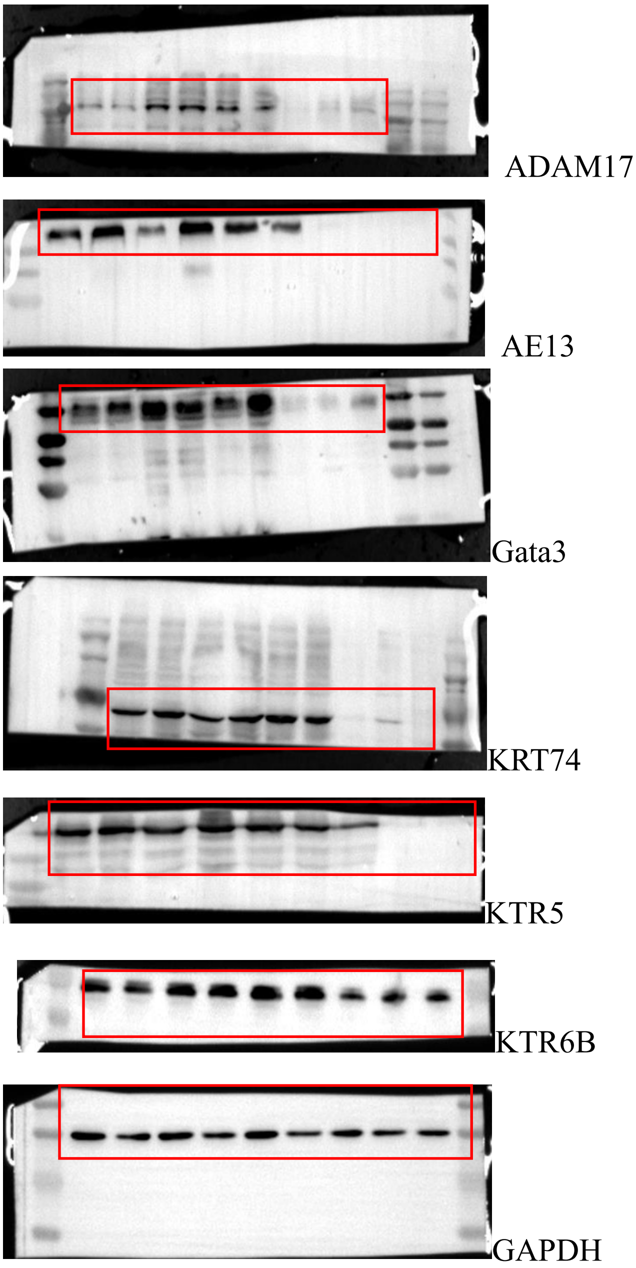

Full unedited gel for Figure 4B

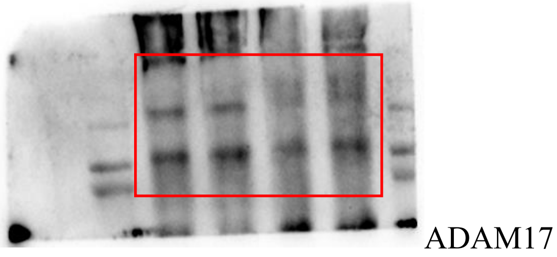

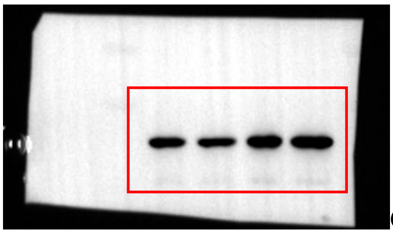

GAPDH

Full unedited gel for Figure 4E

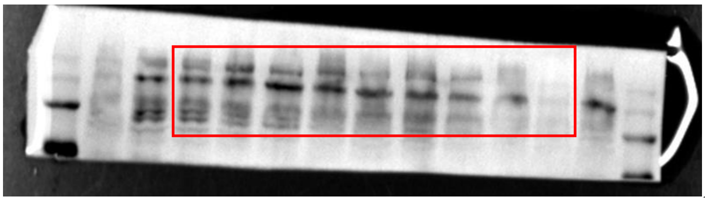

ADAM17

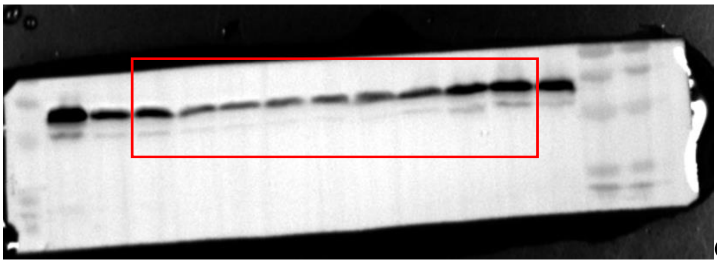

GAPDH

Full unedited gel for Figure 4G

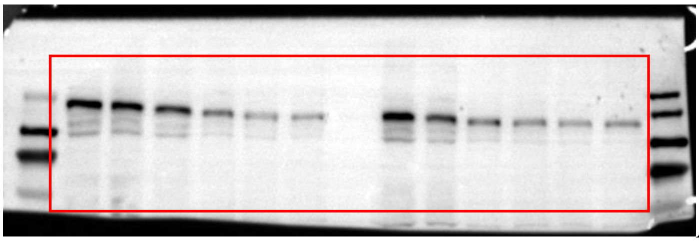

ADAM17

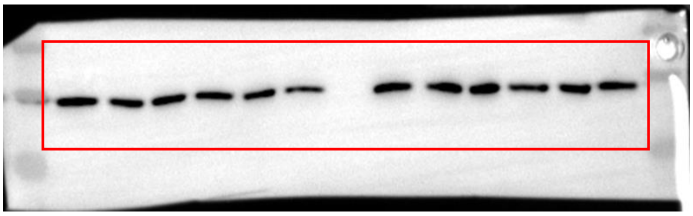

GAPDH

Full unedited gel for Figure 4H

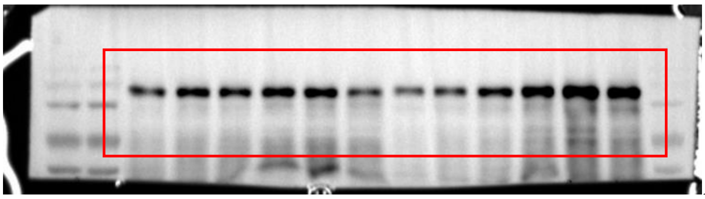

ADAM17

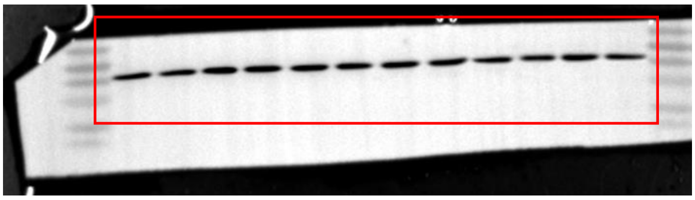

GAPDH

Full unedited gel for Figure 4I

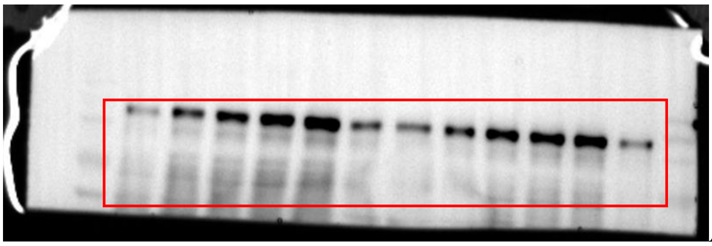

ADAM17

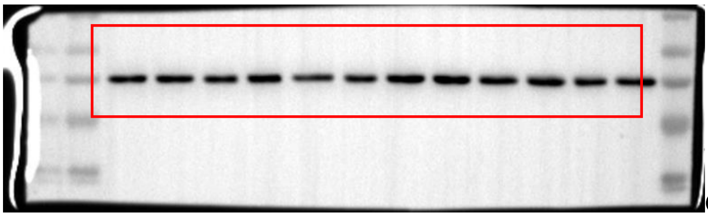

GAPDH

Full unedited gel for Figure 4J

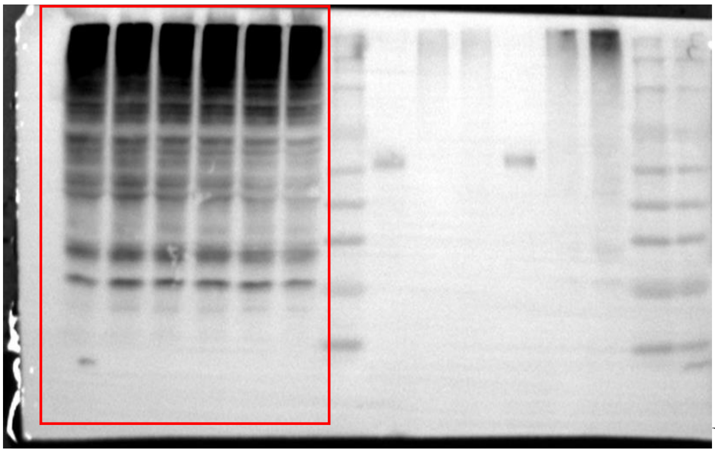

UBQ left panel

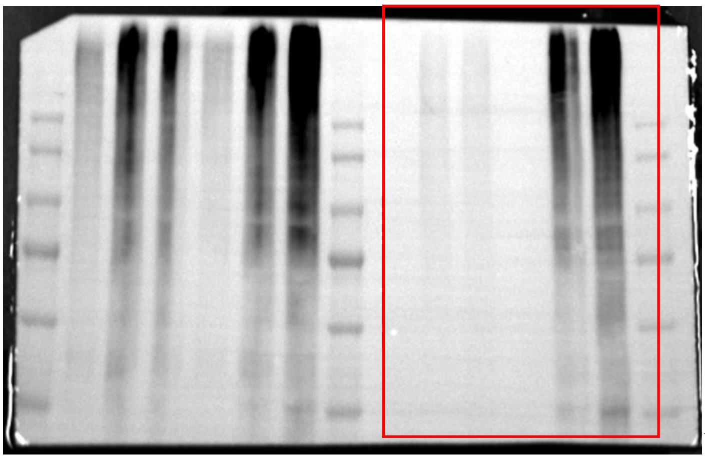

UBQ Right panel

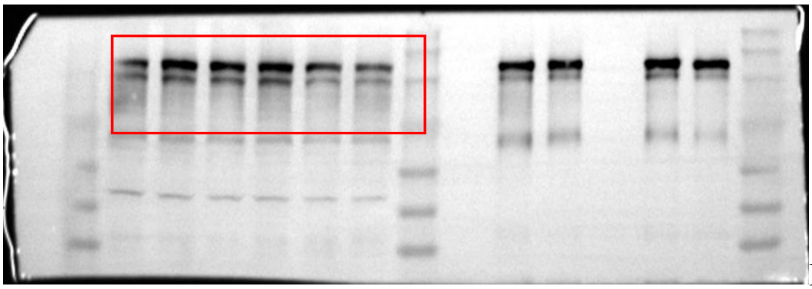

HA left panel

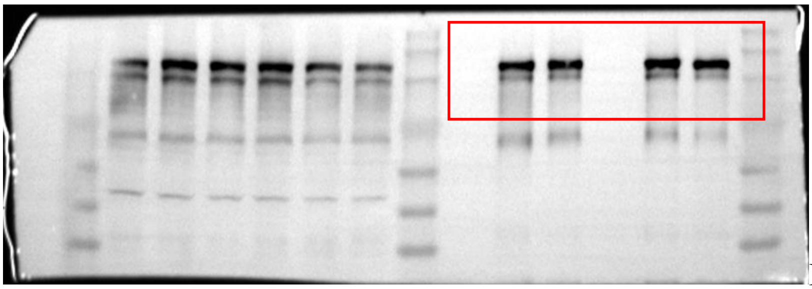

HA Right panel

Full unedited gel for Figure 5C

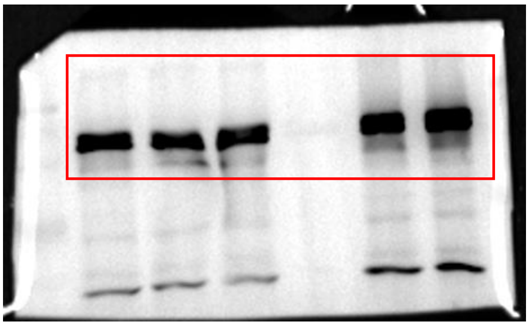

HA tagged ADAM17

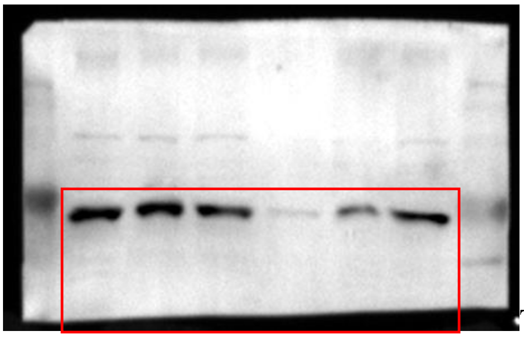

TRIM47

Full unedited gel for Figure 5D

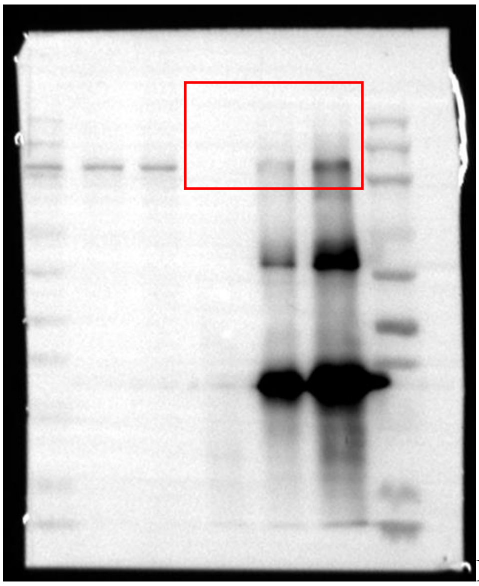
HA-tagged ADAM17

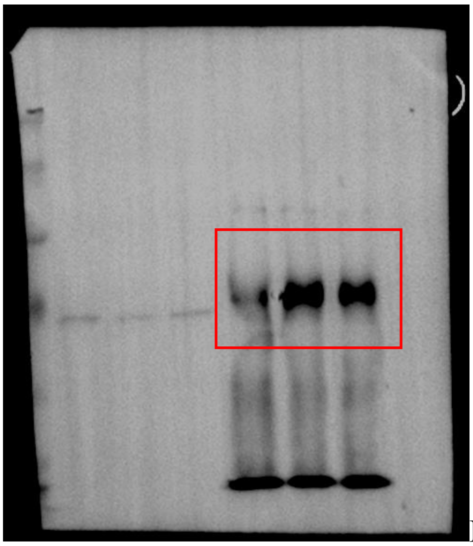
His-tagged TRIM47

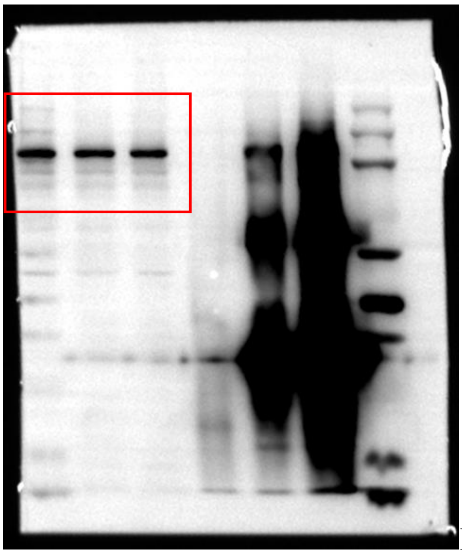
HA-Tagged ADAM17

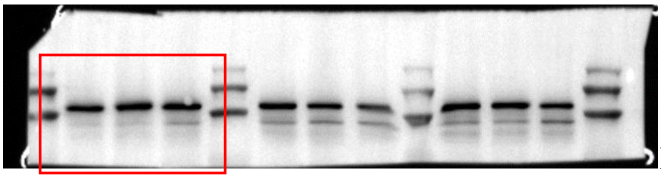

His-tagged TRIM47

Full unedited gel for Figure 5D

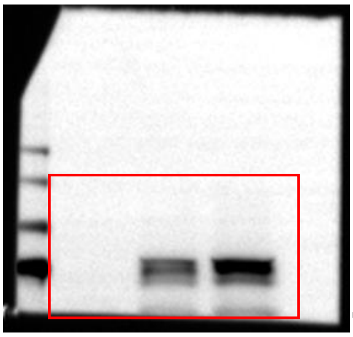

TRIM47

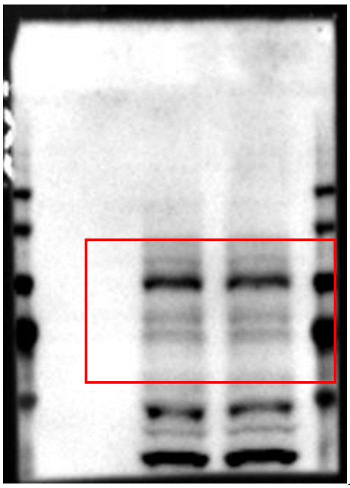

ADAM17

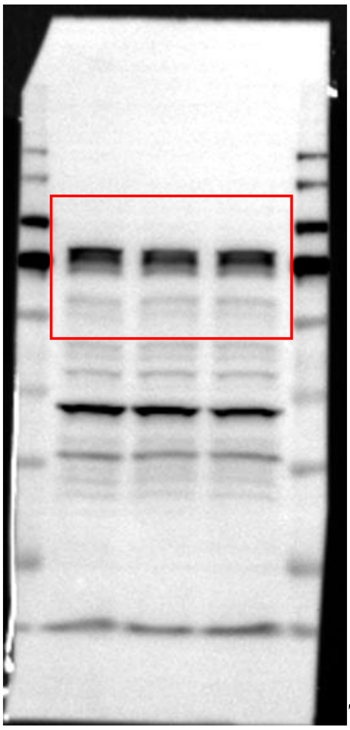

TRIM47

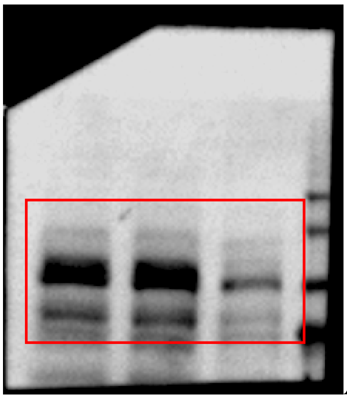

ADAM17

Full unedited gel for Figure 5I

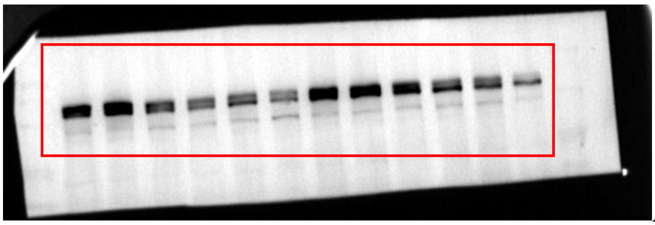

ADAM17

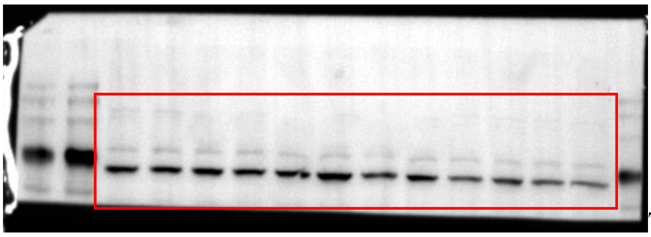

TRIM47

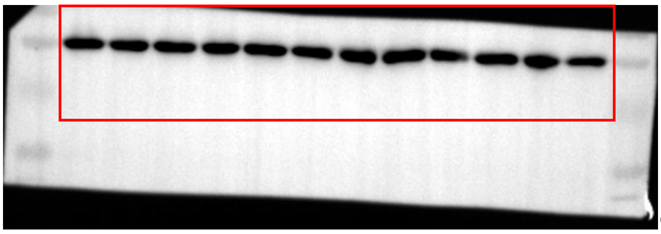

GAPDH

Full unedited gel for Figure 7A

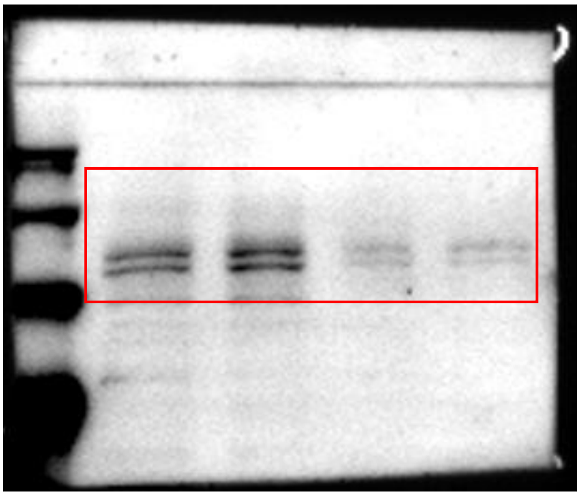

ADAM17

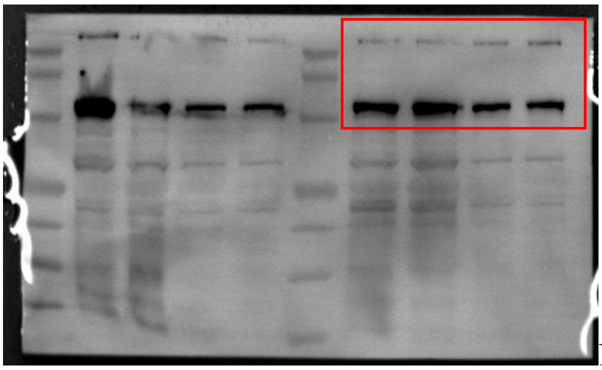

Notch1

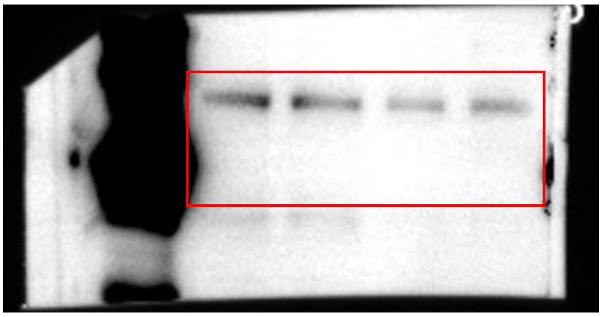

Hes1

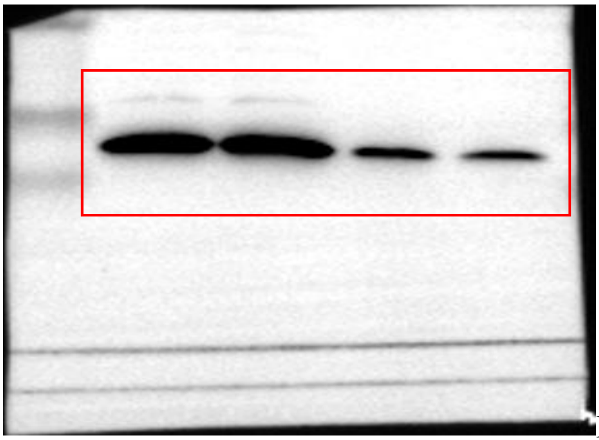
Hes5

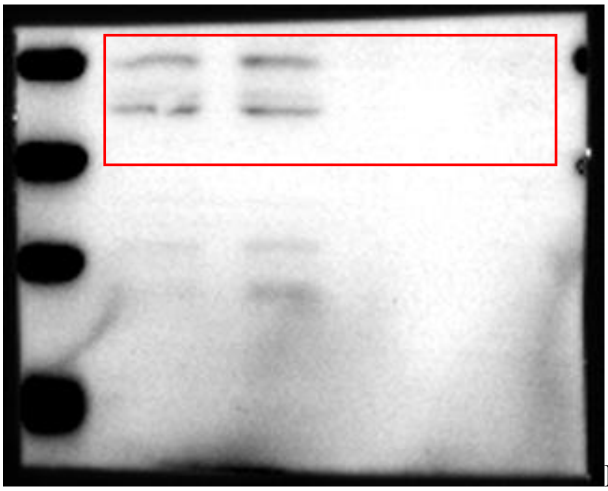
Hey1

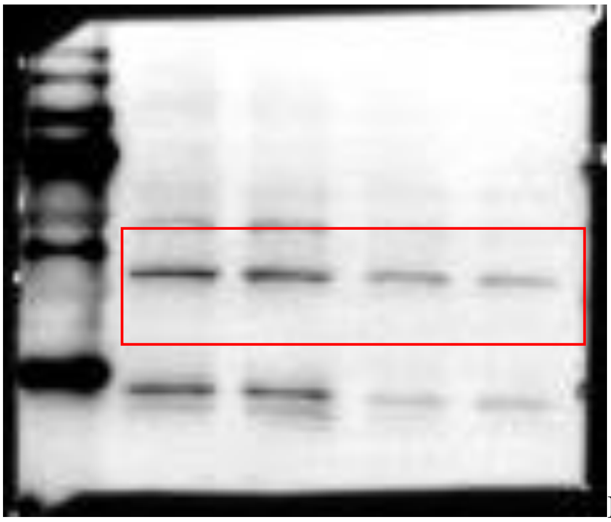
Hey2

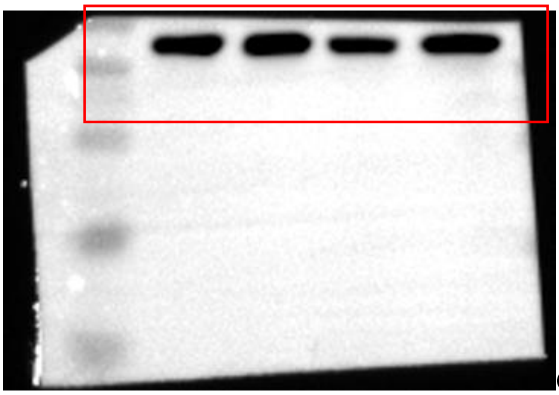

GAPDH

Full unedited gel for Figure 7B

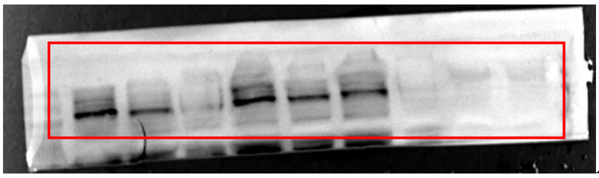

ADAM17

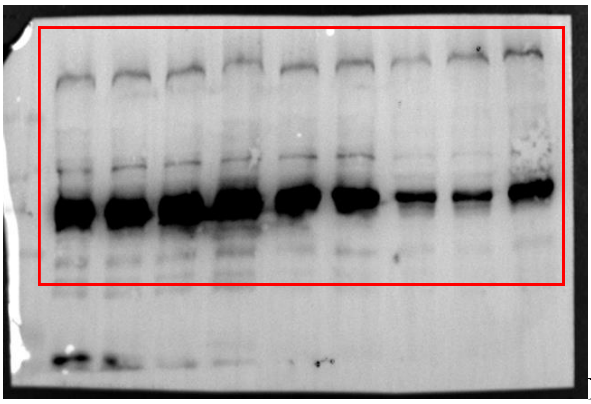

Notch1

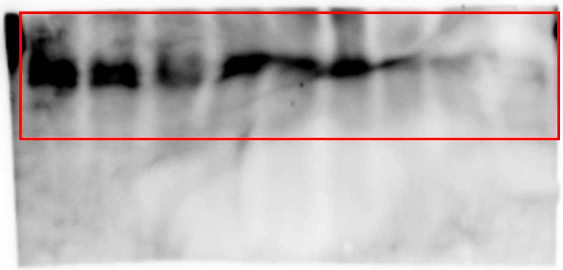

Hes1

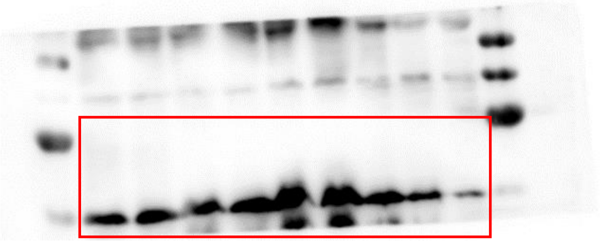

Hes5

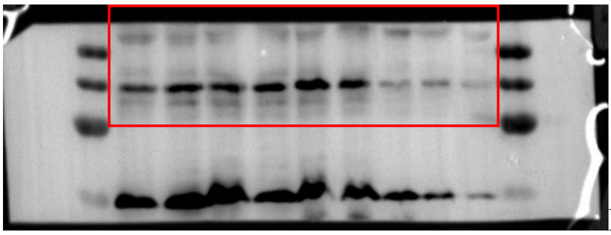
Hey1

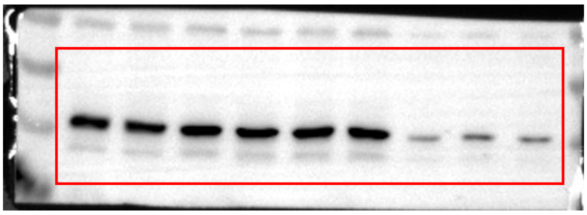
Hey2

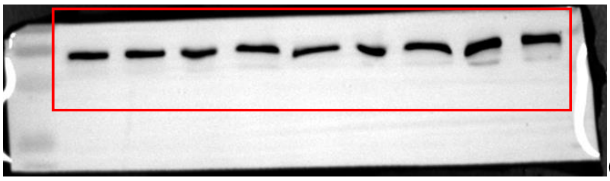
GAPDH

Full unedited gel for Figure 7F

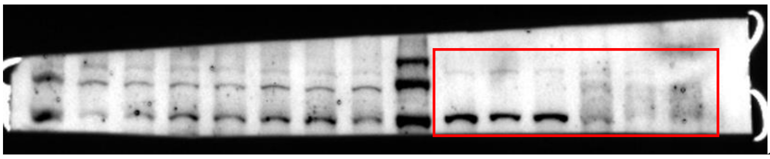
ADAM17

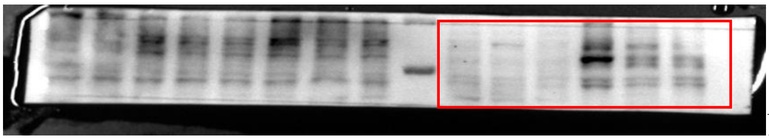
Notch1

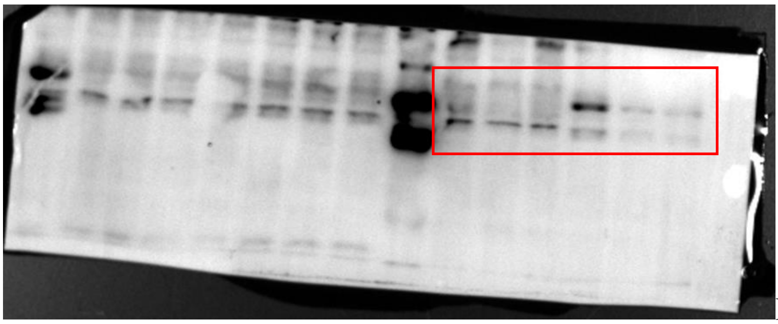
Hes1

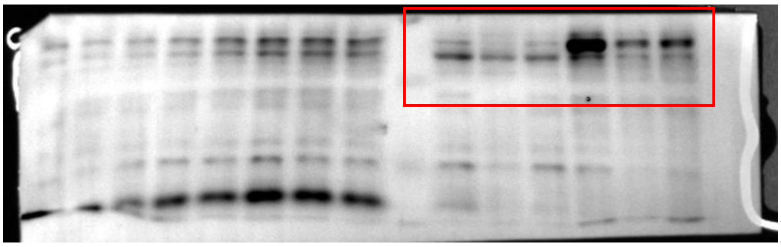
Hes5

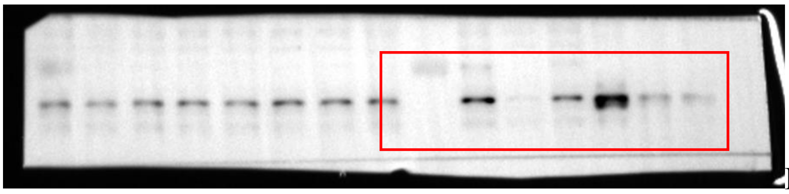

Hey1

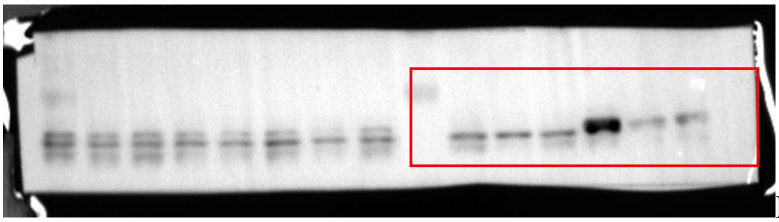

Hey2

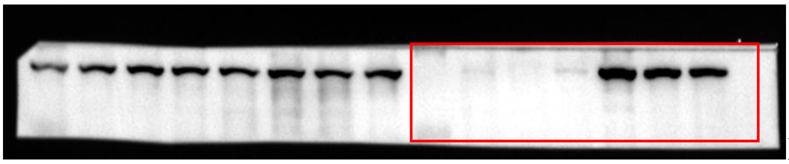

Lamin B

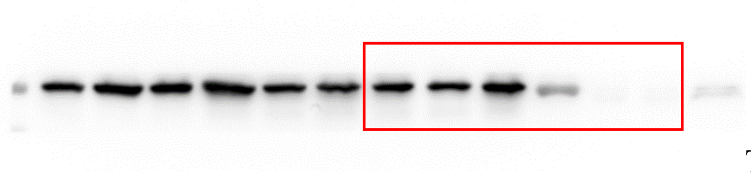

Tubulin

Full unedited gel for Figure 7G

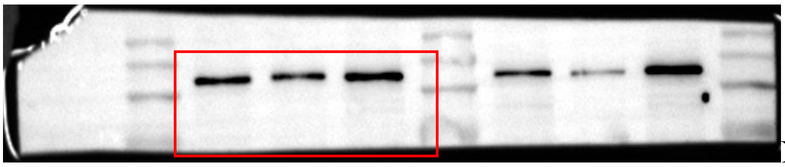

Notch

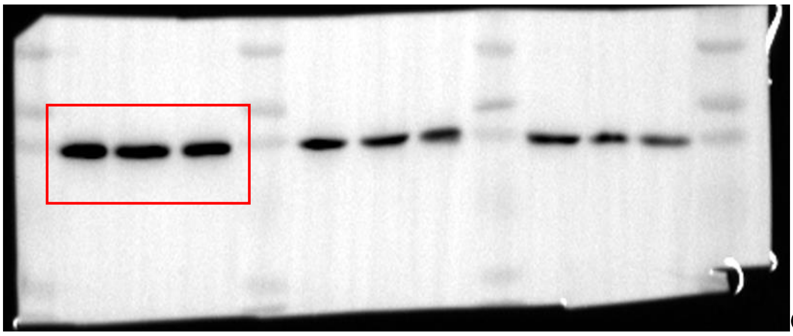

GAPDH

Full unedited gel for Figure S4B

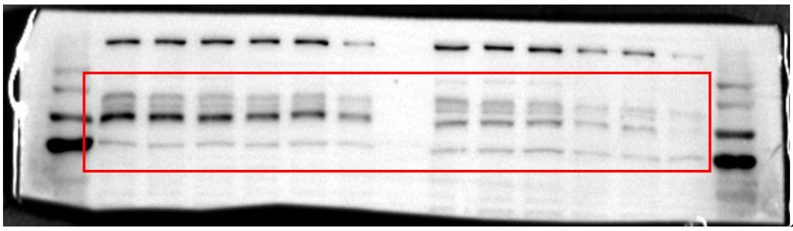

ADAM17

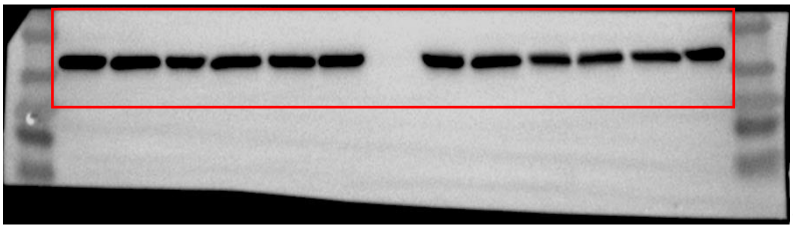

GAPDH

Full unedited gel for Figure S4D

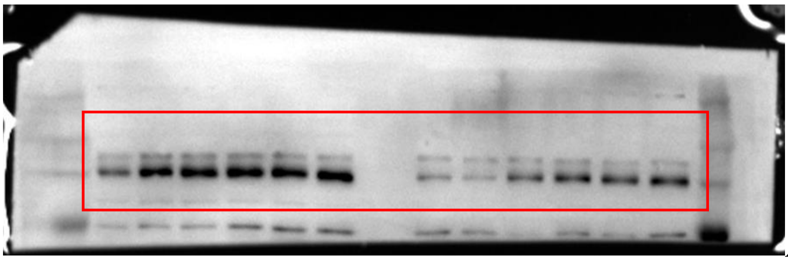

ADAM17

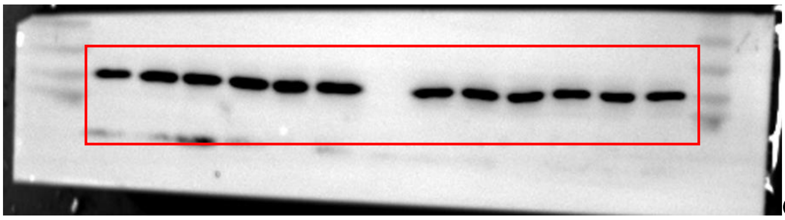

GAPDH

Full unedited gel for Figure S4E

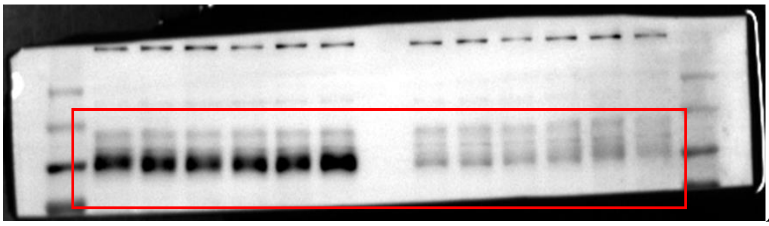

ADAM17

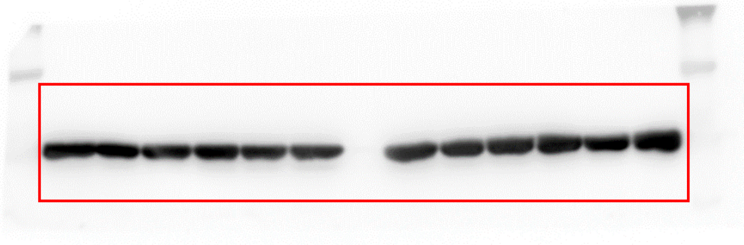

GAPDH

Full unedited gel for Figure S6B

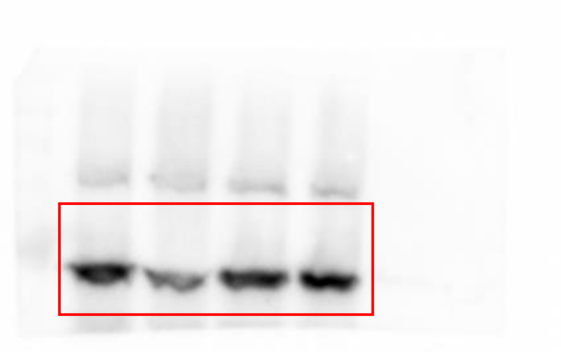

TRIM47

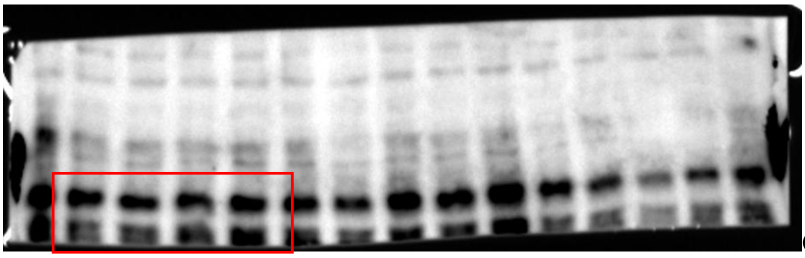

GAPDH

Full unedited gel for Figure S6C

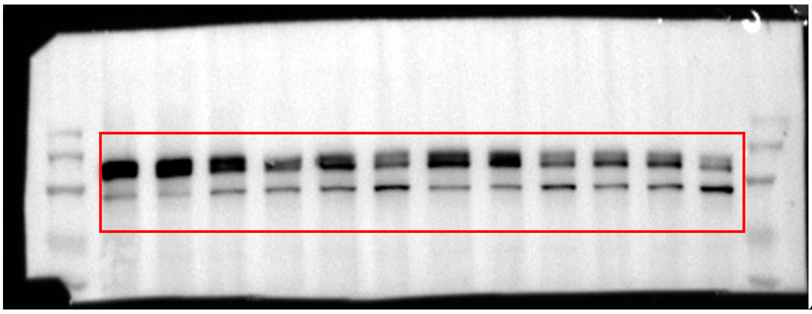

ADAM17

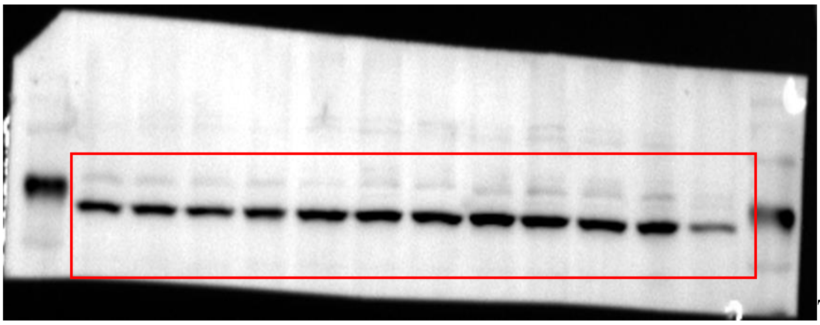

TRIM47

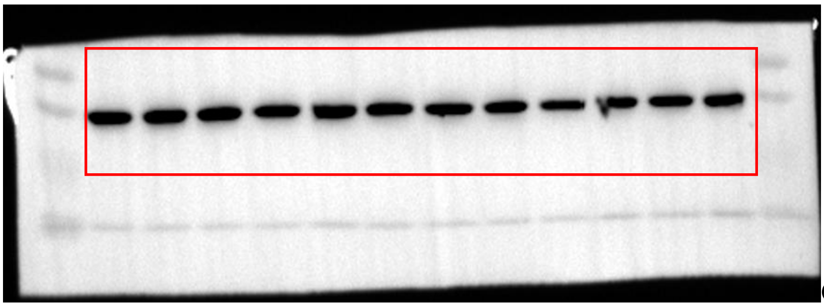

GAPDH

Full unedited gel for Figure S6F

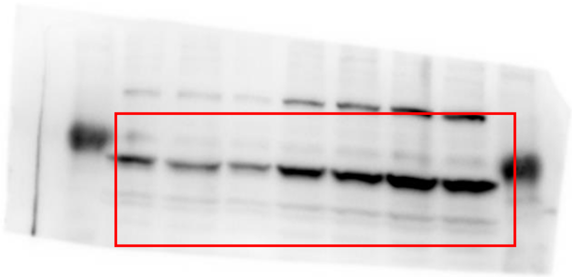

TRIM47

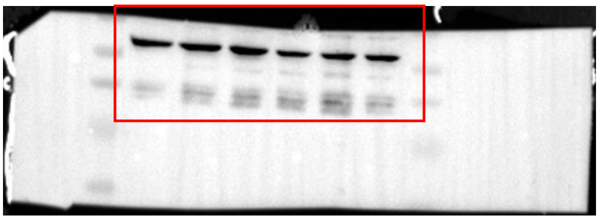

GAPDH

Full unedited gel for Figure S6G

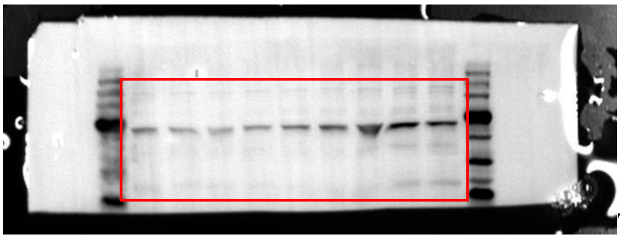

TRIM47

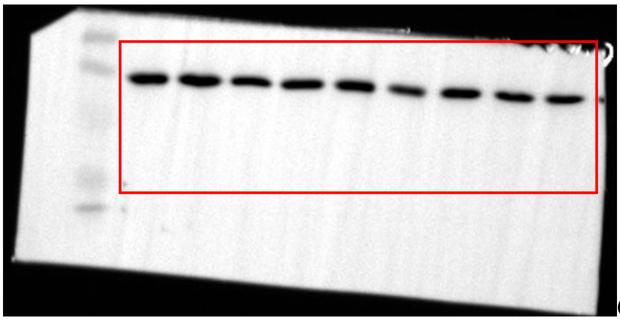

GAPDH
